# Supplementary material for: Gut Microbiota Composition Modulates the Magnitude and Quality of Germinal Centers during Plasmodium Infections
Source: Cell Rep. Author manuscript; Available in PMC 2020 Dec 30. (PMC7772993; doi:10.1016/j.celrep.2020.108503)
Supplement: 1 [file NIHMS1657049-supplement-1.pdf]

Cell Reports, Volume *33*

Supplemental Information

Gut Microbiota Composition Modulates the Magnitude and Quality of Germinal Centers during *Plasmodium* Infections

Morgan L. Waide, Rafael Polidoro, Whitney L. Powell, Joshua E. Denny, Justin Kos, David A. Tieri, Corey T. Watson, and Nathan W. Schmidt

Supplemental Figure 1

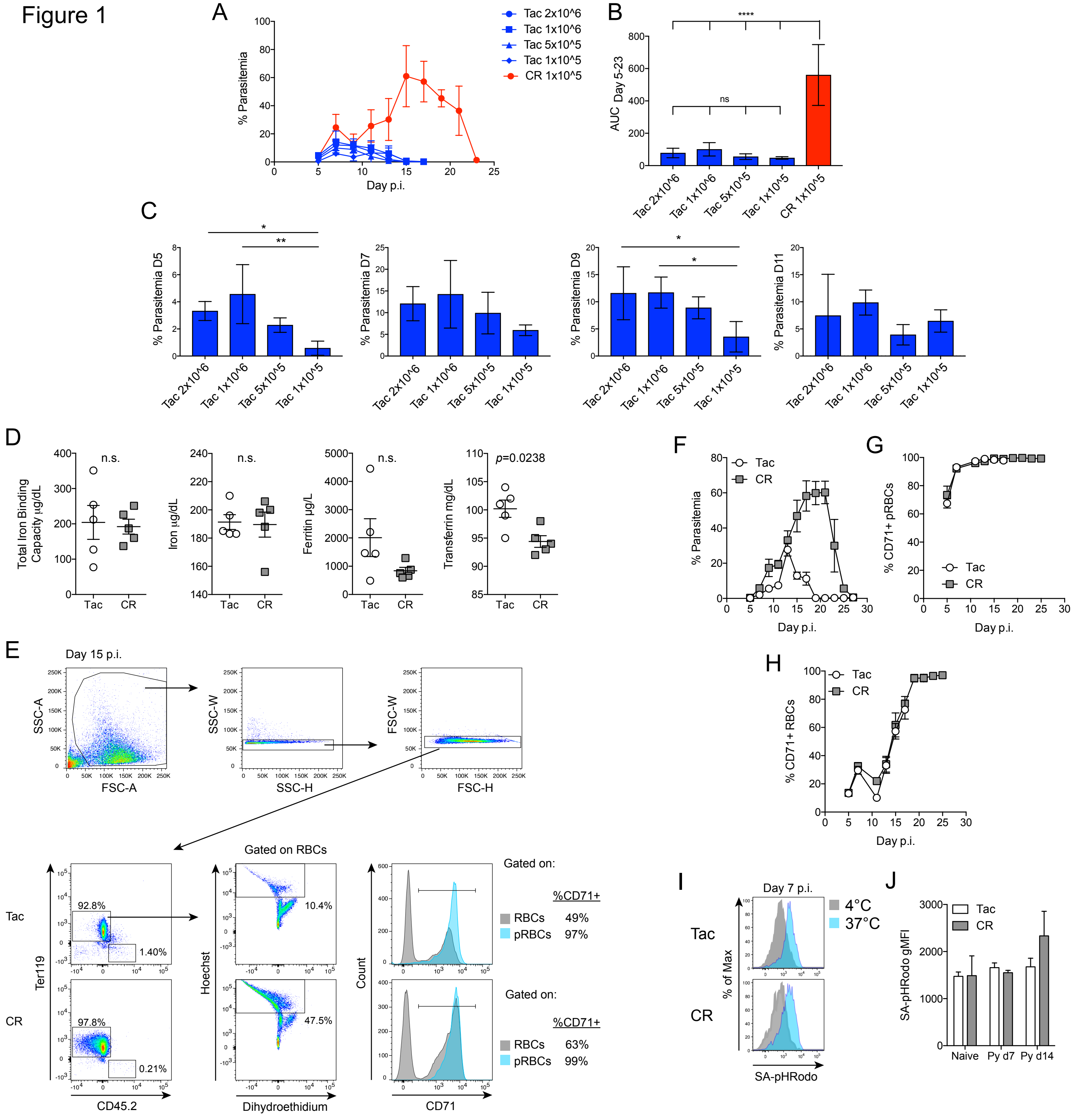

Supplemental Figure 1. Differences in parasite burden not influenced by parasite dose, differential iron levels, erythropoiesis, or macrophage phagocytosis. Related to Figure 1. Resistant Tac mice were infected with increasing doses of *P. yoelii* infected red blood cells. (A) Parasitemia of Tac mice compared to CR mice (n=4). (B) Area under the curve analysis of parasitemia curves. (C) Bar graphs showing parasitemia in Tac mice individually at days 5-11. Data (mean±S.D) were analyzed by One-way ANOVA with multiple comparisons. (D) Results from serum iron panel analysis. Data (mean±S.E.) are from 5 samples (each sample is pooled sera from 2 mice) and were analyzed by Mann-Whitney test. n.s. = not significant. (E-H) Mice from Tac and CR were infected with pRBCs. (E) Representative FACS plots displaying gating strategy for RBCs (Ter-119+, CD45.2-) and pRBCs (Ter-119+, CD45.2-, Hoechst 33342+, dihydroethidium+) that are CD71+. (F) Percent parasitemia. (G) Percent of pRBCs that are CD71+. (H) Percent of total RBCs that are CD71+. Data (mean±S.E.) from 3 mice per group are representative of 3 experiments. (I-J) Phagocytic activity of CD3-CD11bhiCD11c- splenocytes from uninfected or infected mice. (I) Representative histogram of pHrodo® Red expression in CD3-CD11bhiCD11c- splenocytes harvested from day 7 P. yoelii-infected mice following a 1- to 2-hour incubation at the indicated temperature. (J) pHrodo® Red geometric fluorescence intensity (gMFI) in CD3-CD11bhiCD11c- splenocytes after a 1- to 2-hour incubation at 37°C. Data (mean+S.E.) are from 3 mice per group and representative of 2 experiments.

Supplemental Figure 2

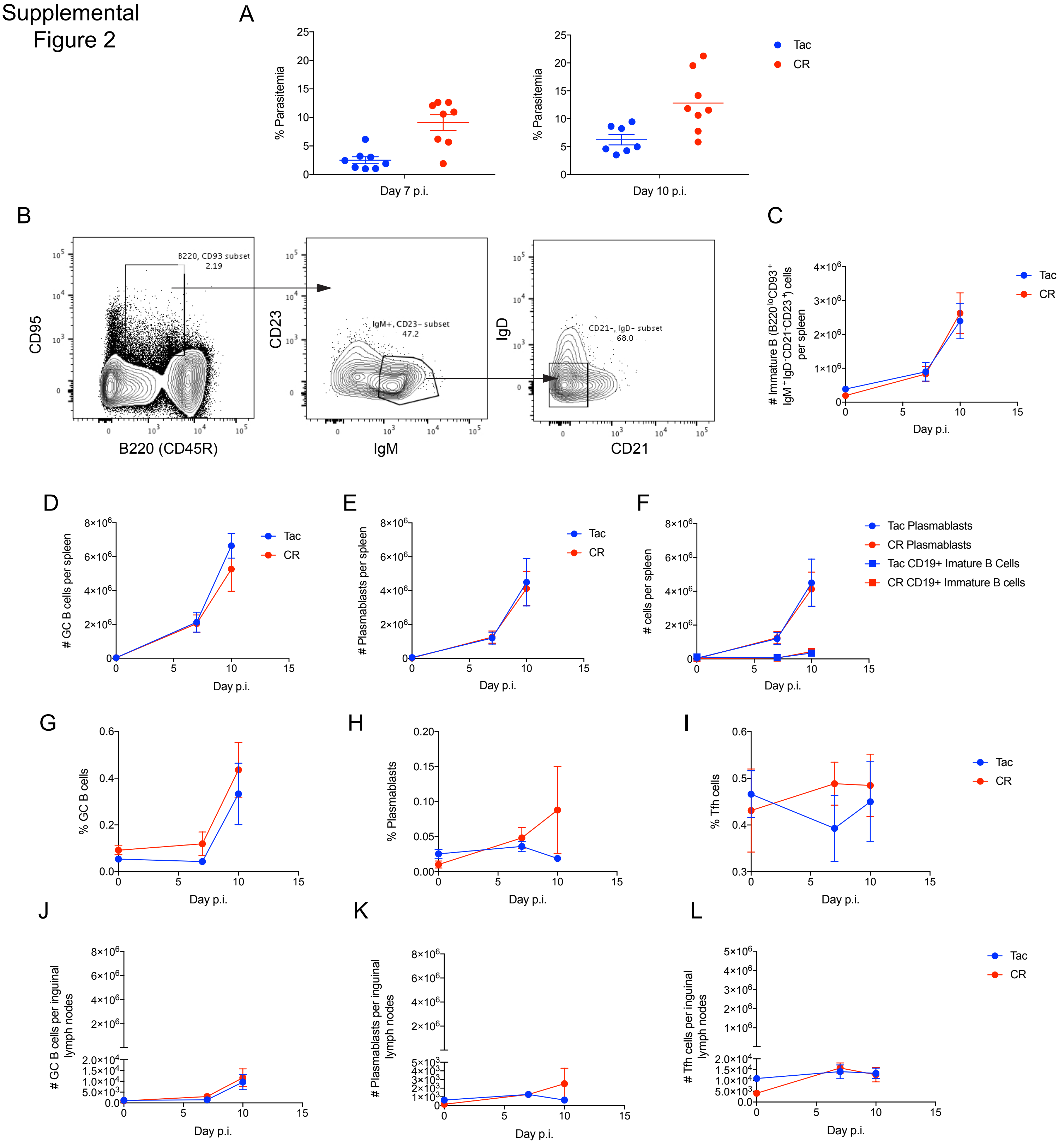

Supplemental Figure 2. Parasite burden in Tac and CR mice is not associated with differential numbers of splenic immature B cells or lymph node Tfh cells, plasmablasts, or GC B cells. Related to Figure 1. Tac and CR mice were infected with *P. yoelii* and spleens and inguinal lymph nodes were removed at days 0, 7, and 10 p.i. (A) Percent parasitemia of mice at the indicated time points. (B) Gating strategy for immature B cells (Ueda, Liao et al. 2007). (C) Numbers of immature B cells (B220loCD93+IgM+IgD-CD21-CD23-), (D) GC B cells (CD19+CD95+GL7+), and (E) plasmablasts (CD19+B220+IgD-CD138+) per spleen. (F) To show that very few immature B cells are incorporated into the plasmablasts staining panel, immature B cells were gated on CD19 and the total number of CD19+ immature B cells and Plasmablasts per spleen were shown on the same graph. (D-I) The frequencies and total cell numbers of Tfh cells (G, J), Plasmablasts (H,K), and GC B cells (I, L) in the inguinal lymph nodes are shown. Data (mean±S.E.) are from two independent experiments with n=8 mice per timepoint.

Supplemental Figure 3

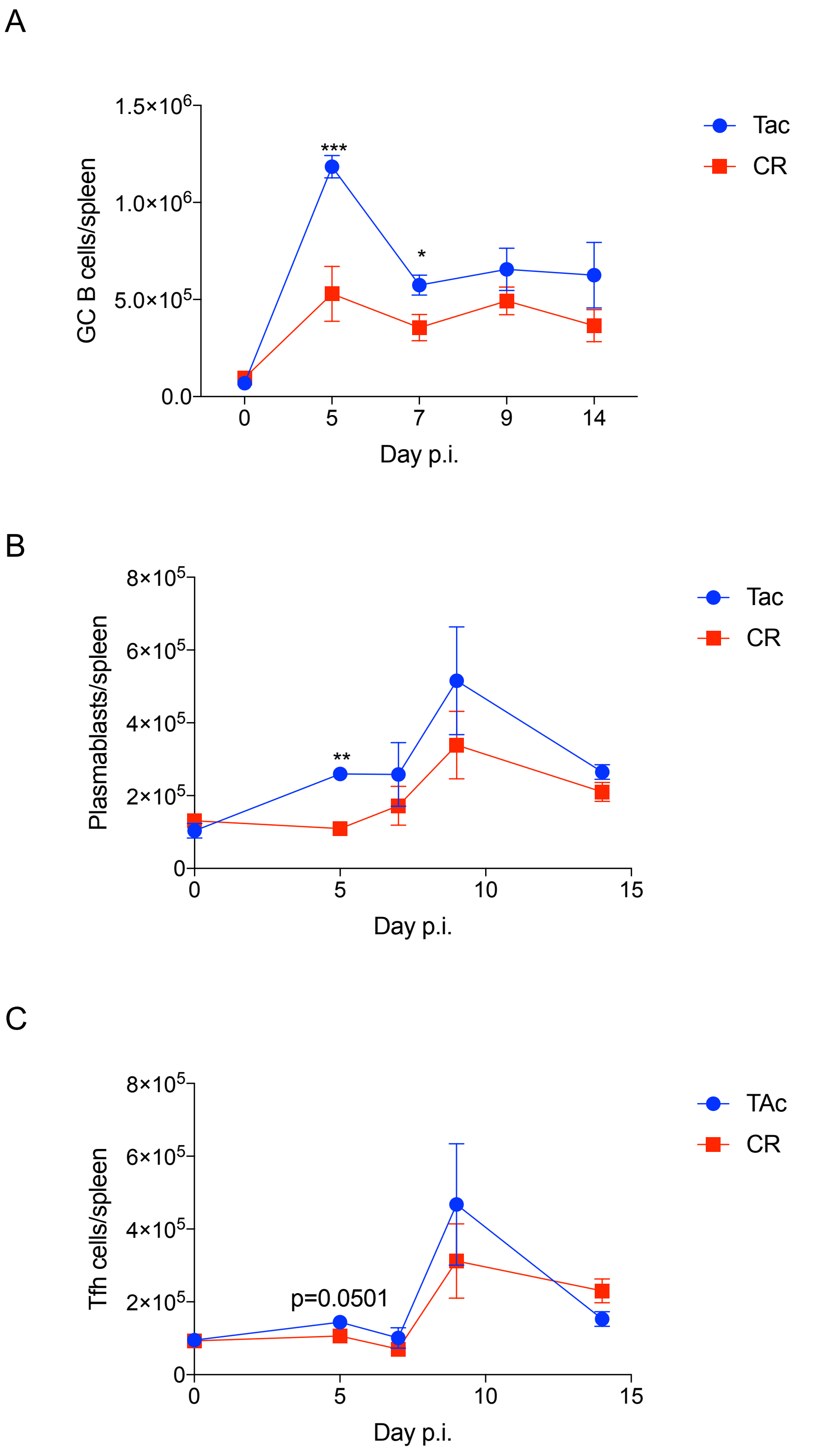

Supplemental Figure 3. Tac mice immunized with sheep red blood cells exhibit increased GC B cell response compared to immunized CR mice. Related to Figure 1. Tac and CR mice received a single I.P. injection of 150 uL citrated sheep red blood cells. Spleens were removed at days 0, 5, 7, 9, and 14. GC B cell (A), plasmablast (B), and Tfh cell (C) numbers per spleen were analyzed. Data (mean±S.E.) are from two independent experiments (n=3-7 mice per timepoint) and were analyzed by unpaired T test * $p < 0.05$, ** $p < 0.01$, *** $p < 0.001$.

Supplemental Figure 4

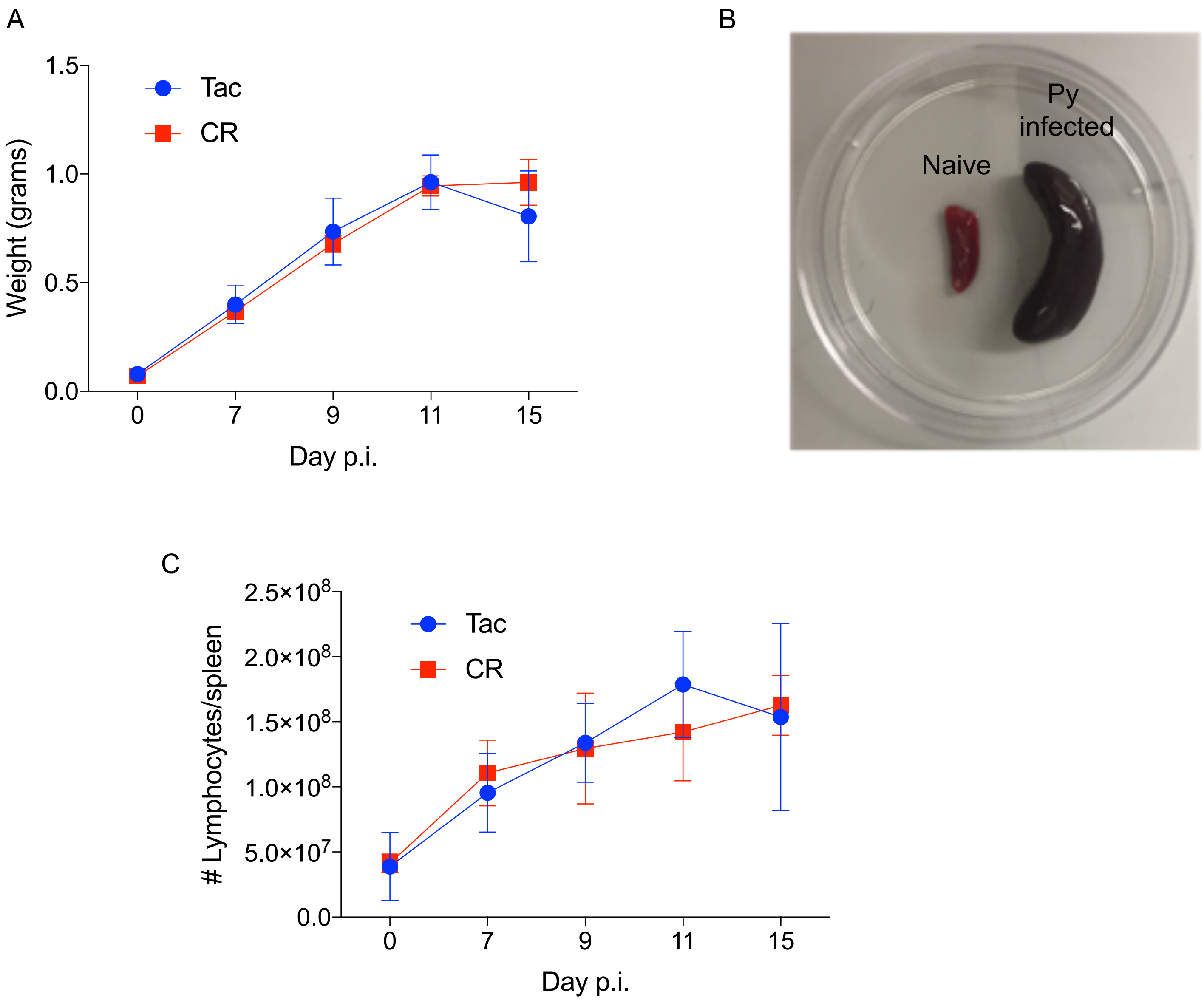

Supplemental Figure 4. Splenic expansion occurs equally in both resistant and susceptible mice. Related to Figure 3. Spleens were removed from both Tac and CR mice between days 0 and 15 post *P. yoelii* infection. (A) Spleen weights were recorded at each timepoint. (B) Comparison of naïve spleen (left) compared to infected spleen (right) to illustrate change in size and color post *Plasmodium* infection. (C). Number of lymphocytes per spleen were calculated using a hemocytometer at each timepoint. Data (mean+S.E.) are from 3 separate experiments.

Supplemental Figure 5

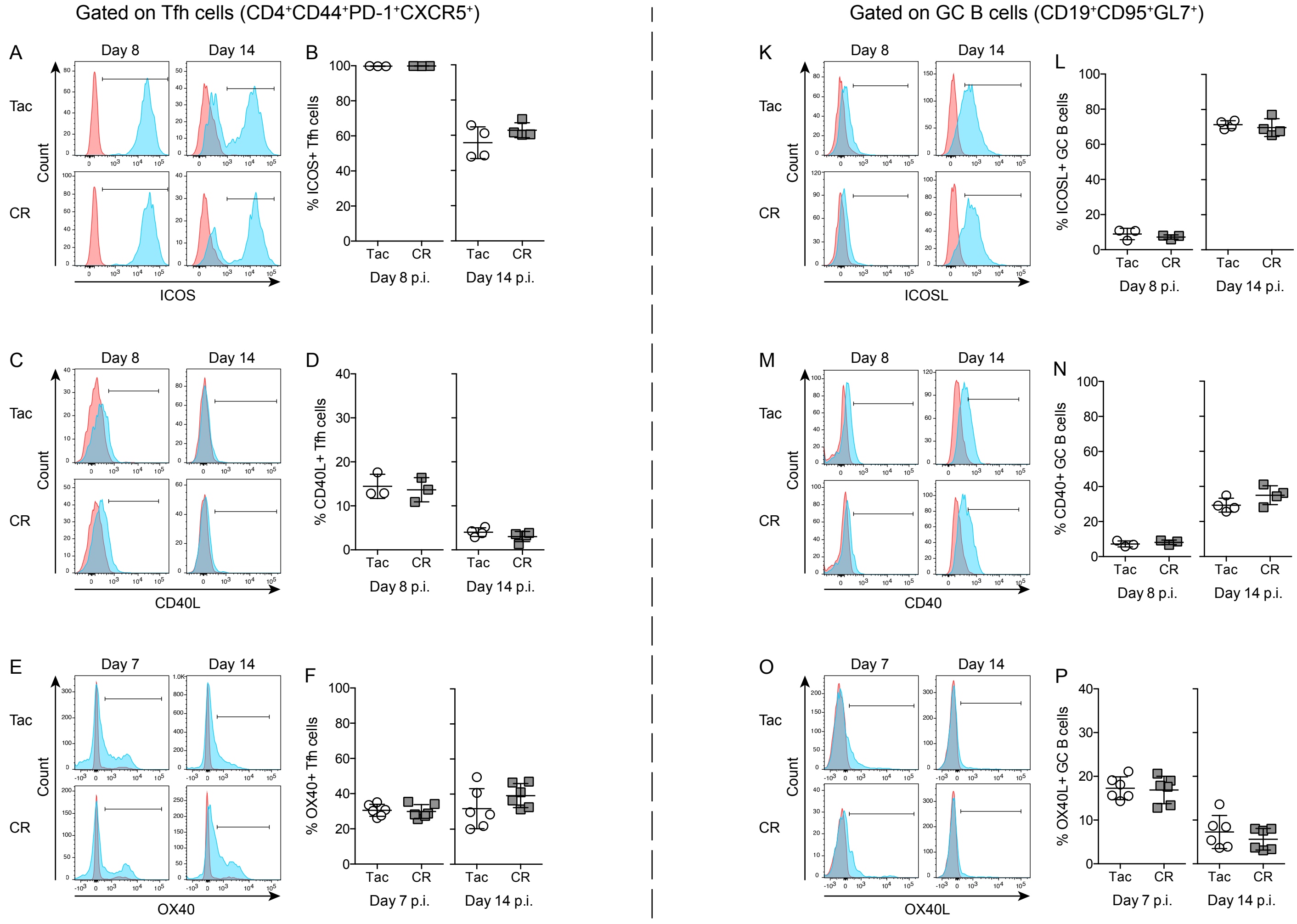

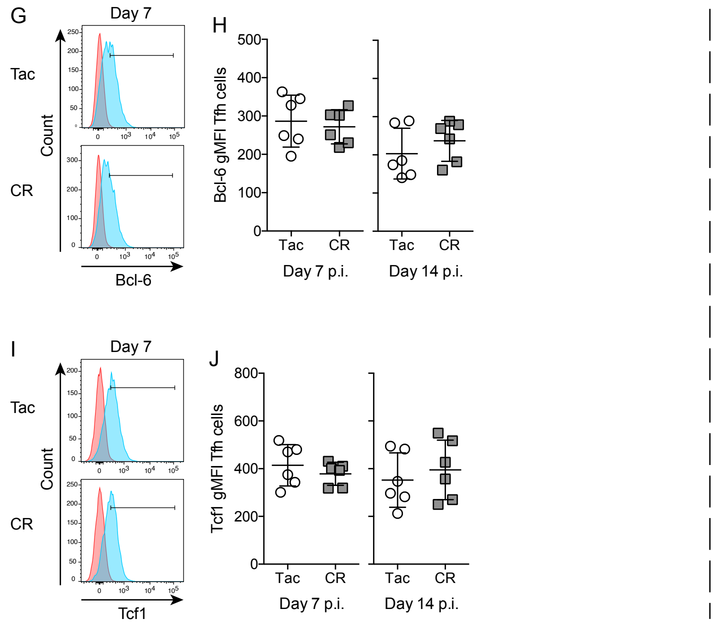

Supplemental Figure 5. Phenotypic analysis of co-stimulatory receptors and transcription factors on Tfh cells and GC B cells. Related to Figure 4. Tac and CR mice were infected with *P. yoelii* and spleens were removed on the indicated day post infection. Flow cytometry analysis on Tfh cells (CD4+CD44+PD-1+CXCR5+) for ICOS (A-B), CD40L (C-D), OX40 (E-F), Bcl-6 (G-H), or TCf1 (I-J). Flow cytometry analysis on GC B cells (CD19+CD95+GL7+) for ICOSL (K-L), CD40 (M-N), or OX40L (O-P). Data (mean+S.E.) are from 1 to 2 experiments.

Supplemental Figure 6

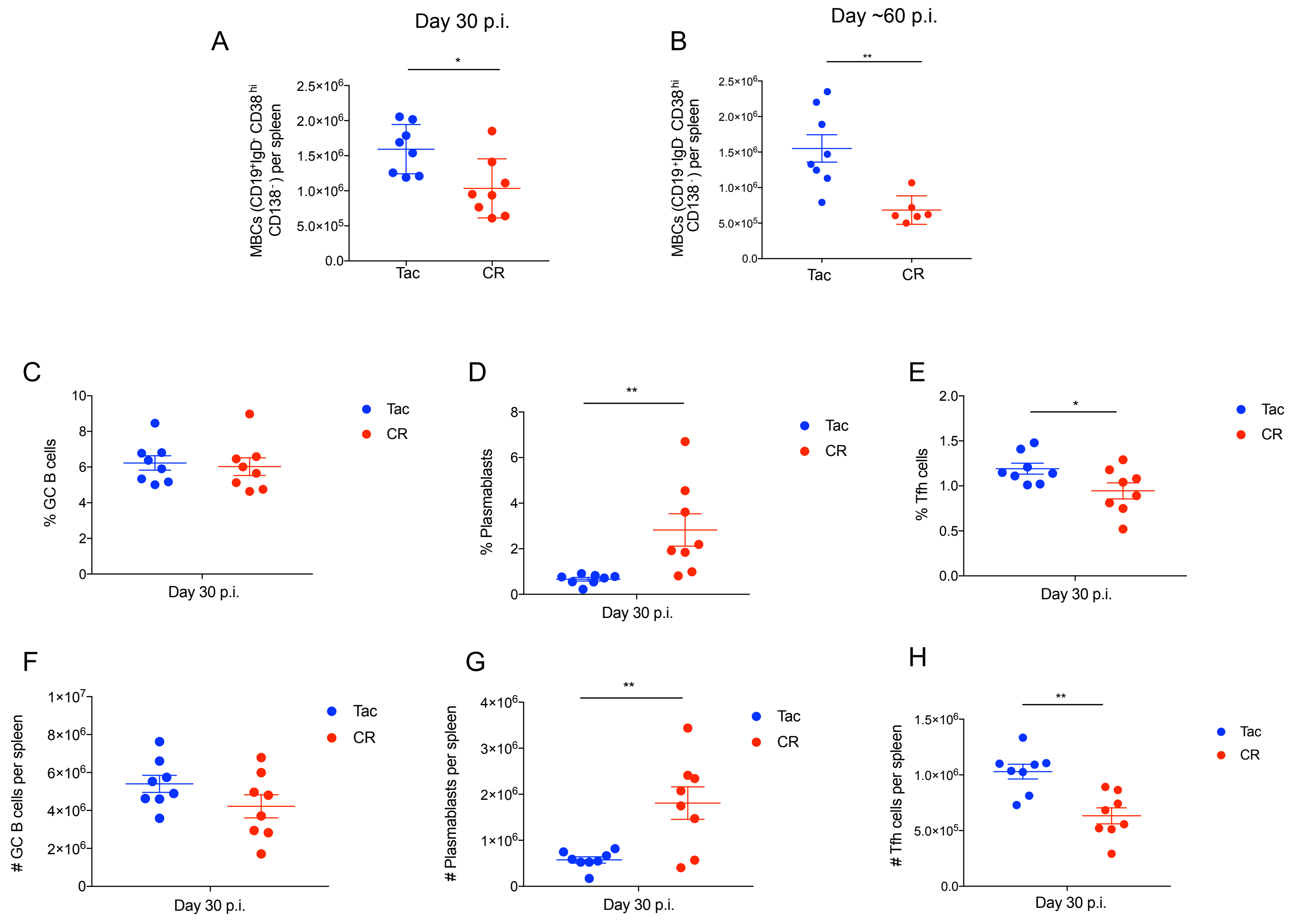

Supplemental Figure 6. Cellular analysis in the spleens of mice post *Plasmodium* clearance. Related to Figure 4. Tac and CR mice were infected with *P. yoelii*. Spleens were removed and cellular analysis done following clearance. The number of memory B cells (CD19+IgD-CD38hiCD138-) cells per spleen were determined at (A) day 30 and (B) day 60-62 p.i. The frequency and number of (C, F) GC B cells, (D, G) plasmablasts, and (E, H) Tfh cells were determined at day 30 post infection. Data (mean+S.E.) are from two separate experiments with n=6-8 mice per timepoint and were analyzed by unpaired T test * $p < 0.05$, ** $p < 0.01$

Supplemental Figure 7

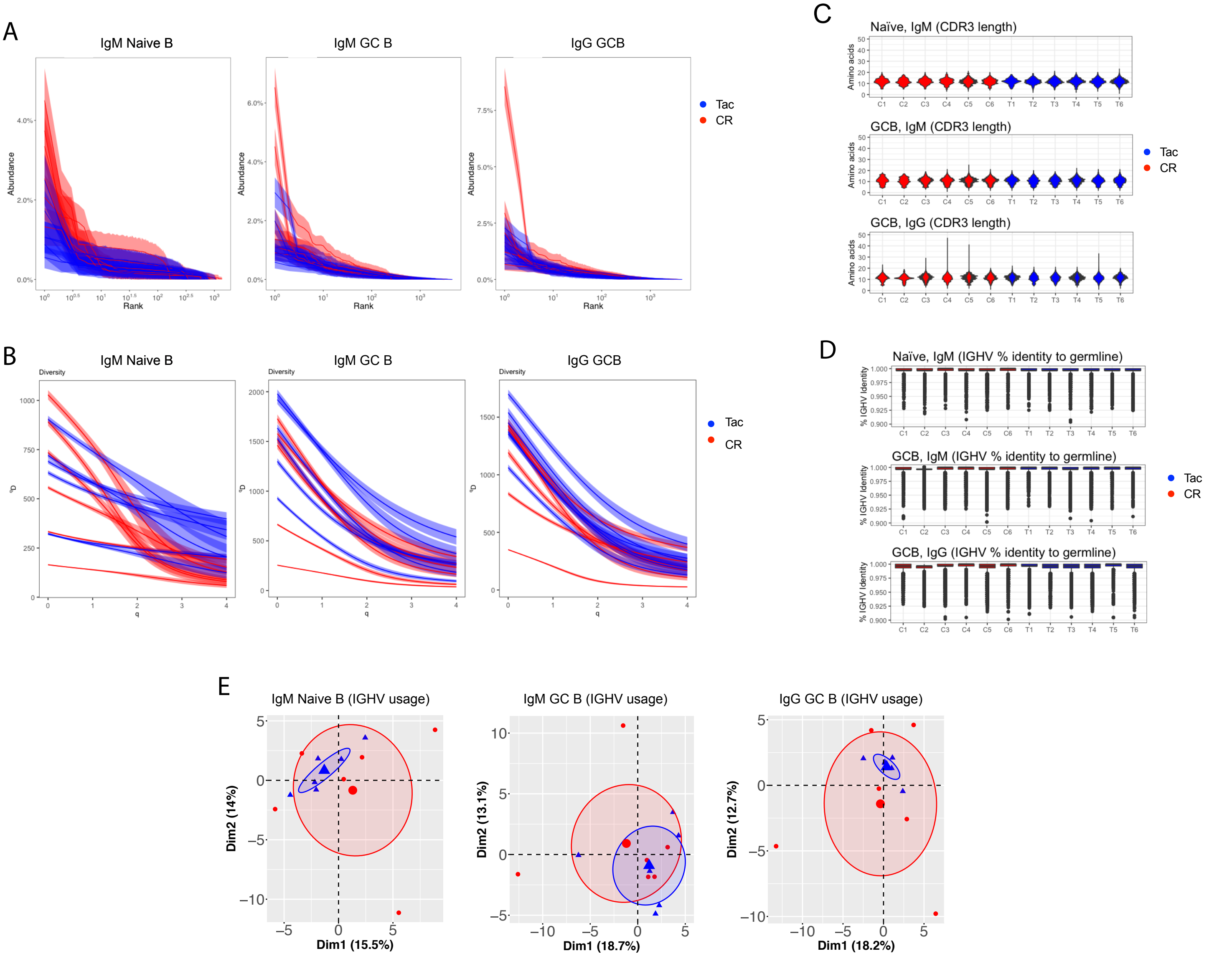

Supplemental Figure 7. Additional BCR analysis in Tac and CR naïve and GC B cells. Related to Figure 5. (A) Clonal abundance curves and (B) repertoire diversity curves at diversity orders, q=0-4, are shown for each animal for naïve cells, IgM+ GC B cells, and IgG+ GC B cells. Shading in plots represents the 95% confidence interval, as determined by 200 bootstrap resamplings of the data. (C) Variation in CDR3 amino acid length across all sequences in naïve cells, IgM+ GC B cells, and IgG+ GC B cells. (D) Variation in percent identity matches of each IGHV sequence to the closest germline IGHV gene. (E) Plots of the first two principle components associated with variation in IGHV gene usage in naïve cells, IgM+ GC B cells, and IgG+ GC B cells, each point representing an individual animal. Ellipses representing the 95% confidence interval around the centroid (large symbol) of each group are shown. Data for each animal are colored by group (red, CR, n=6; blue, Tac, n=6).

Supplemental Table 1. Repertoire sequencing library metrics, related to Figure 5. Repertoire sequencing library metrics for 6 Tac (T) mice and 6 CR (C) mice at the processing step of the analysis. Samples were processed end-to-end using tools/software within the Immcantation pipeline.

Sample	Subset	Isotype	Mask Primers	Filter Length	Collapse Seq
T1	GCB	IGM	287666	223545	87107
T1	GCB	IGG	566741	436494	150478
T1	N	IGM	98251	66908	20729
C1	GCB	IGM	181121	118500	40185
C1	GCB	IGG	235058	118377	41536
C1	N	IGM	135413	76497	24977
T2	GCB	IGM	224432	161007	54695
T2	GCB	IGG	328378	184456	63304
T2	N	IGM	258843	95458	28974
C2	GCB	IGM	234222	154403	41644
C2	GCB	IGG	224955	115217	33442
C2	N	IGM	143852	73206	20383
T3	GCB	IGM	644981	441600	151621
T3	GCB	IGG	480006	275387	100028
T3	N	IGM	217858	125158	41708
C3	GCB	IGM	871160	639836	223103
C3	GCB	IGG	708422	451758	157817
C3	N	IGM	131315	85139	31476
T4	GCB	IGM	1086665	702395	248925
T4	GCB	IGG	1015353	707352	247355
T4	N	IGM	706524	140944	46025
C4	GCB	IGM	528872	345868	124013
C4	GCB	IGG	183988	112431	45607
C4	N	IGM	323325	118636	38285
T5	GCB	IGM	278999	142960	55883
T5	GCB	IGG	141823	79810	32975
T5	N	IGM	968299	175663	56509
C5	GCB	IGM	1887600	1299232	439461
C5	GCB	IGG	1665908	964155	314222
C5	N	IGM	540189	266160	88439
T6	N	IGM	475287	228067	76141
C6	N	IGM	203445	83307	29945
T6	GCB	IGM	333549	211314	81241
T6	GCB	IGG	448507	255978	96530
C6	GCB	IGM	994200	639063	230509
C6	GCB	IGG	324214	176141	70687

Supplemental Table 2. (See data in excel document). Results of one-way ANOVA, related to Figure 5. Results of one-way ANOVA, assessing differential usage of IGHV and IGHJ gene between Tac and CR mice in naïve cells, IgM+ GC B cells, and IgG+ GC B cells. Both uncorrected P values and q values based on the false-discovery rate (Benjamini and Hochberg 1995) are provided.
