# Supplementary material for: Gut Microbiota Composition Modulates the Magnitude and Quality of Germinal Centers during Plasmodium Infections
Source: Cell Rep. Author manuscript; Available in PMC 2020 Dec 30. (PMC7772993; doi:10.1016/j.celrep.2020.108503)
Supplement: 3 [file NIHMS1657049-supplement-3.pdf]

Cell Reports

Article

Gut Microbiota Composition Modulates the Magnitude and Quality of Germinal Centers during *Plasmodium* Infections

Graphical Abstract

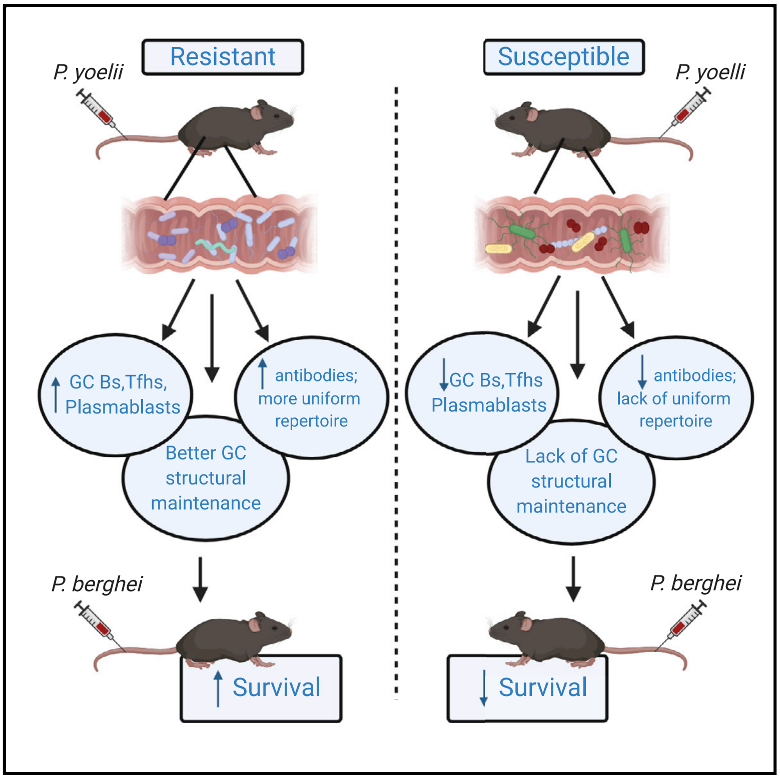

Highlights

- Gut microbiota influence *Plasmodium*-induced spleen GC cells and maintenance
- Gut microbiota effect quantity and quality of *Plasmodium*-specific antibodies
- Gut microbiota modulation of GCs is sufficient to affect the severity of malaria
- Microbiota regulation of 1° immune response affects outcome of *Plasmodium* challenge

Authors

Morgan L. Waide, Rafael Polidoro, Whitney L. Powell, ..., David A. Tieri, Corey T. Watson, Nathan W. Schmidt

Correspondence

nwschmid@iu.edu

In Brief

Research has shown that gut microbiota composition influences malaria severity, but the mechanism has remained unclear. Waide et al. show that microbiota composition drives differences in the humoral immune response, including differences in germinal center cell numbers and parasite-specific antibodies, ultimately affecting the memory response to subsequent infection.

Waide et al., 2020, Cell Reports *33*, 108503
December 15, 2020 © 2020 The Author(s).
https://doi.org/10.1016/j.celrep.2020.108503

CellPress

Cell Reports

CellPress OPEN ACCESS

Article

Gut Microbiota Composition Modulates the Magnitude and Quality of Germinal Centers during *Plasmodium* Infections

Morgan L. Waide,[1,2] Rafael Polidoro,[2] Whitney L. Powell,[1] Joshua E. Denny,[1] Justin Kos,[3] David A. Tieri,[3] Corey T. Watson,[3] and Nathan W. Schmidt[1,2,4,*]

[1]Department of Microbiology and Immunology, University of Louisville, Louisville, KY, USA

[2]Ryan White Center for Pediatric Infectious Diseases and Global Health, Department of Pediatrics, Indiana University School of Medicine, Indianapolis, IN, USA

[3]Department of Biochemistry, University of Louisville, Louisville, KY, USA

[4]Present address: Ryan White Center for Pediatric Infectious Diseases and Global Health, Department of Pediatrics, Indiana University School of Medicine, Indianapolis, IN 46202, USA

*Correspondence: nwschmid@iu.edu

https://doi.org/10.1016/j.celrep.2020.108503

SUMMARY

Gut microbiota composition is associated with human and rodent *Plasmodium* infections, yet the mechanism by which gut microbiota affects the severity of malaria remains unknown. Humoral immunity is critical in mediating the clearance of *Plasmodium* blood stage infections, prompting the hypothesis that mice with gut microbiota-dependent decreases in parasite burden exhibit better germinal center (GC) responses. In support of this hypothesis, mice with a low parasite burden exhibit increases in GC B cell numbers and parasite-specific antibody titers, as well as better maintenance of GC structures and a more targeted, qualitatively different antibody response. This enhanced humoral immunity affects memory, as mice with a low parasite burden exhibit robust protection against challenge with a heterologous, lethal *Plasmodium* species. These results demonstrate that gut microbiota composition influences the biology of spleen GCs as well as the titer and repertoire of parasite-specific antibodies, identifying potential approaches to develop optimal treatments for malaria.

INTRODUCTION

Plasmodium infections led to an estimated 228 million cases of malaria and 405,000 deaths in 2018 (World Health Organization, 2019). Although >40% of the world's population live in areas affected by malaria, there is currently no long-term, effective vaccine and resistance to antimalarial and prophylaxis drugs is continuing to spread (Ashley et al., 2014; Phyo et al., 2016; Menard and Dondorp, 2017; Hamilton et al., 2019; van der Pluijm et al., 2019) while efforts to decrease the incidence of malaria and deaths have stalled (World Health Organization, 2018). Malaria poses a significant health risk worldwide, with an economic impediment reaching an estimated US$12 billion/year owing to clinical costs, distributing antimalarial drugs, and the distribution of other preventive measures (Gallup and Sachs, 2001; Nonvignon et al., 2016). This illustrates the growing need for novel, inexpensive, and easily deployable treatment options.

Resistance to *Plasmodium* infection can be acquired in individuals living in endemic regions, but only after a period of years and repeated exposures. This delayed resistance is evident in the higher incidence of severe disease and mortality in young children (Marsh and Kinyanjui, 2006). The role of the humoral immune system in *Plasmodium* clearance was demonstrated when the transfer of sera from immune individuals into infected children resulted in reduced parasite burden (Cohen et al., 1961). It has since been shown that this acquired resistance correlates with humoral immunity, but antibody responses in children are typically short-lived and long-term resistance is the result of years of gradually increasing *Plasmodium*-specific antibodies (Crompton et al., 2010; Weiss et al., 2010), which includes a combination of increased numbers of *Plasmodium*-specific antibodies in circulation along with an increased breadth of antigens that these antibodies recognize (Osier et al., 2008; Nogaro et al., 2011). It is known that there is large interindividual variation in the antibody response following *Plasmodium* infection (Burel et al., 2017). Importantly, it is not presently known whether gut microbiota composition contributes to interindividual variation in antibody responses or whether it affects the magnitude and repertoire of *Plasmodium*-specific antibodies.

T follicular helper (Tfh) cells are necessary for the germinal center (GC) reaction and provide signals for GC B cells to develop into long-lived antibody-producing plasmablasts and memory B cells (MBCs; Crotty, 2014). Previous studies in mouse models have shown that in the absence of properly activated Tfh cells, there is a complete disruption of the GC response and thus an inability to clear *Plasmodium* (Pérez-Mazliah et al., 2017).

This is an open access article under the CC BY license (http://creativecommons.org/licenses/by/4.0/).

Furthermore, interleukin-21 (IL-21) production in Tfh cells is necessary for GC B cell responses, and mice deficient in IL-21 signaling are unable to resolve *Plasmodium* infection (Pérez-Mazliah et al., 2015). The importance of IL-21 signaling in the development of B cell responses in human *Plasmodium* infections has likewise been confirmed (Figueiredo et al., 2017). In *Plasmodium*-infected children, a Th1 polarized response negatively affects the differentiation of GC B cells, and the presence of Th1-associated interferon-γ (IFN-γ) suppresses GC responses in mice (Obeng-Adjei et al., 2015; Guthmiller et al., 2017). These data demonstrate the necessity of an efficient humoral immune response to clear *Plasmodium* infections and that future malaria treatments and vaccine efforts should target the development of a robust GC response.

Gut microbiota affect a range of physiologic functions (Shreiner et al., 2015; Durack and Lynch, 2019), including immune system development and protection against infection and disease (Kamada et al., 2013; Kamada and Núñez 2014; Nguyen et al., 2016; Kim and Kim, 2017). Prior studies have shown that gut bacteria and the metabolites they produce affect Th17 and regulatory T cell (Treg) responses, driving differences in the balance of proinflammatory responses and immune system regulation (Arpaia et al., 2013; Lécuyer et al., 2014). A particular member of the mouse gut microbiota, segmented filamentous bacteria (SFB), has been shown to induce the differentiation of Tfh cells in the small intestine Peyer's patches. These cells then egress to gut-distal lymphoid tissues to induce GC responses and autoantibody production (Teng et al., 2016). This ability of gut microbiota to influence T cell differentiation contributes to numerous extra-gastrointestinal (GI) immune disorders (asthma, allergies, and eczema) and autoimmune diseases (autoimmune arthritis, type 1 diabetes, and experimental autoimmune encephalomyelitis) (Sprouse et al., 2019). In addition to T cell responses, gut microbiota-driven cytokine production influences the differentiation of regulatory B cells in secondary lymphoid tissues such as the spleen and mesenteric lymph nodes (Rosser et al., 2014). Previous studies have also shown a role for Toll-like receptor 5 sensing of gut bacteria-derived flagellin and microbial metabolites such as short-chain fatty acids on plasma cell development and the antibody response to the trivalent seasonal influenza vaccine and *Citrobacter rodentium* infection, respectively (Oh et al., 2014; Kim et al., 2016). However, we have a limited understanding of how gut microbiota modulates host immunity—in particularly B cell responses—to extra-GI tract infections, such as *Plasmodium*. The limited information regarding the impact of gut microbiota on humoral immunity to extra-GI infections (influenza and lymphocytic choriomeningitis virus [LCMV]) was generated in mice treated with broad-spectrum antibiotics (Ichinohe et al., 2011 Abt et al., 2012). These studies made the important observation that gut microbiota are required for efficient immune responses to systemic viral infections. Still, the use of a cocktail of broad-spectrum antibiotics to generate mice largely devoid of gut bacteria precludes our knowledge of whether and how unique compositions of gut microbial communities that are observed in individuals affects host immunity to extra-GI infections.

Evidence points to a potential role for the gut microbiota in the interaction with human and rodent *Plasmodium* species and risk of infection (Yilmaz et al., 2014; Yooseph et al., 2015). We have previously shown that gut microbiota composition influences severe malaria in mice infected with *Plasmodium yoelii* 17XNL (Villarino et al., 2016). These differences in gut microbiota composition between vendors and the resulting severity—a measurement of the length of infection and peak parasitemia typically depicted through area under the parasitemia curve analysis (Villarino et al., 2016)—of *Plasmodium* infection has remained consistent in subsequent experiments (Denny et al., 2019). Gut microbiota-induced resistance to severe malaria in these mice correlates with increased humoral immune responses (Villarino et al., 2016), yet it remains unknown whether this or other mechanisms are responsible for gut microbiota-dependent modulation of *Plasmodium* infection. This observation led to the desire to explicitly test the hypothesis that the composition of the gut microbiota affects humoral immunity to *Plasmodium* through regulating GC biology, thus affecting the quantity and quality of the resulting *Plasmodium*-specific antibodies. In support of this hypothesis, we show here that mice resistant to *Plasmodium* infection have gut microbiota-dependent increases in GC-associated immune cells and *Plasmodium*-specific antibody titers. Furthermore, resistant mice maintain better GC architecture, have signatures of increased diversity in their antibody repertoires, and maintain increased long-term resistance to *Plasmodium* challenge.

RESULTS

Gut Microbiota Composition Causes Differential GC Reactions following *P. yoelii* Infection That Correlate with Resistance to Hyperparasitemia

It has been shown that C57BL/6 mice ordered from different vendors exhibit profound differences in their susceptibility to *P. yoelii* 17XNL infection due to differences in gut microbiota composition (Villarino et al., 2016; Denny et al., 2019). To better characterize the differences in the humoral immune response between resistant and susceptible mice, spleens and sera were collected from mice between days 0 and 21 post-infection (p.i.) (Figure 1). Taconic Biosciences (Tac) C57BL/6N mice, which in our experiments typically show a *P. yoelii* hyperparasitemia-resistant phenotype (Villarino et al., 2016; Denny et al., 2019), and Charles River (CR) C57BL/6N mice, which in our experiments typically show a *P. yoelii* hyperparasitemia-susceptible phenotype (Villarino et al., 2016; Denny et al., 2019), were infected with *P. yoelii* 17XNL infected red blood cells (RBCs) and the percentage of parasitemia was evaluated on the indicated days (Figure 1A). Differences in parasite burden between Tac and CR mice were 2-fold or less through day 14 post infection (p.i.); thereafter Tac mice were able to clear the infection whereas CR mice display increasingly high parasitemia with delayed clearance kinetics (Figure 1A). Of note, this profound difference in parasite kinetics does not seem to be a result of differences in infectious dose or early parasite burden, as infecting Tac mice with 20-fold more parasites minimally increases parasitemia through day 9 p.i. without affecting clearance kinetics or total parasite burden (Figures S1A–S1C). Because of this divergence in parasitemia (day 14 p.i.; Figure 1A) around the onset of the adaptive immune response and the critical role of GC-dependent antibody

CellPress
OPEN ACCESS

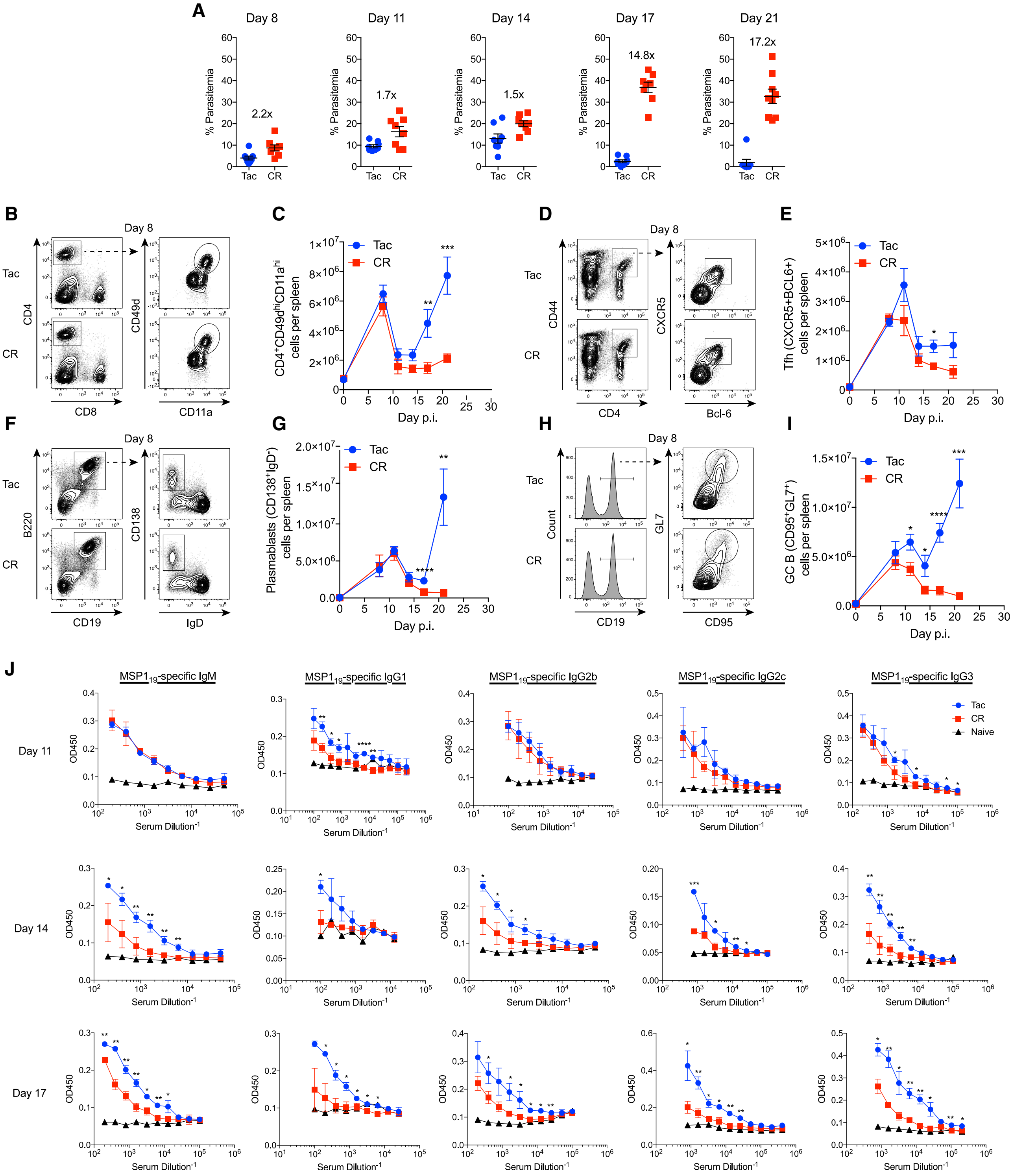

Figure 1. Resistant Tac Mice Exhibit Increased Expansion of GC B Cells and Increased Titers of Parasite MSP1-Specific Antibodies Preceding Clearance of *P. yoelii*

C57BL/6N mice from Taconic Biosciences (Tac) and Charles River (CR) Laboratories were infected with *P. yoelii* parasitized red blood cells (pRBCs).

(A) Percentage of RBCs infected with *P. yoelii* (% parasitemia) on the indicated day post-*P. yoelii* infection. Data (means ± SEs) are cumulative results from 3 experiments. Numbers within each day are the fold difference between the mean % parasitemia in Tac and CR mice.

(legend continued on next page)

production for the clearance of *Plasmodium*-infected RBCs (Blackman et al., 1990; Hill et al., 2013), we hypothesized that resistant mice would exhibit increased numbers of antigen-specific CD4 T cells, Tfh cells, plasmablasts, and GC B cells. Consistent with the hypothesis, cellular analysis revealed that resistant Tac mice experience a significant increase in antigen-experienced CD4 T cells (Figures 1B and 1C), Tfh cells (Figures 1D and 1E), plasmablasts (Figures 1F and 1G), and GC B cells (Figures 1H and 1I) at later stages of the infection. Notably, an increase in GC B cells was seen at days 11 and 14 p.i., suggesting that GC B cells, in particular, may play a role in the divergence of parasitemia observed after day 14 (Figure 1I).

Consistent with a similar parasite burden between Tac and CR mice on day 11 p.i., circulating merozoite surface protein 1_{19} ($MSP1_{19}$)-specific antibody titers showed minimal differences between Tac and CR mice at day 11 p.i. (Figure 1J). In contrast, $MSP1_{19}$-specific antibody titers were significantly higher in Tac mice on days 14 and 17 p.i. (Figure 1J), which correlates with day 14 p.i. as the divergent point in parasitemia between Tac and CR mice (Figure 1A). These data are congruent with the increase in GC B cells observed on day 11 p.i. and thereafter, which subsequently result in increased parasite-specific antibodies and control of parasitemia. Of note, no differences were seen between other factors that may influence parasite burden such as iron levels (higher iron levels support higher parasitemia), erythropoiesis (Py 17XNL prefers to infect reticulocytes), and macrophage phagocytosis (Figures S1D–S1J), providing further support that the observed differences in GC B cells and antibody titers are likely responsible for the differential parasite burden. Likewise, there were no differences in Tfh cells, plasmablasts, and GC B cell frequencies or numbers in the lymph nodes and no differences in splenic immature B cell numbers (Figure S2). Importantly, Tac mice also exhibited an increase in GC B cells following sheep RBC immunization compared to CR mice (Figure S3), demonstrating that these differences in GC responses between Tac and CR mice are not unique to *Plasmodium* infections. Collectively, these data suggest that gut microbiota may function through GC reactions to control the severity of malaria.

To evaluate whether differences in GC reactions between Tac and CR mice are gut microbiota dependent, genetically identical C57BL/6 germ-free (GF) mice were colonized with cecal contents from either Tac (GF+Tac) or CR (GF+CR) mice. As shown previously (Villarino et al., 2016), these colonized mice exhibited the same parasitemia kinetics as control Tac and CR mice (Figures 2A and 2B). Spleens from colonized GF mice and control mice were removed at day 19 p.i. and greater numbers of antigen-experienced CD4 T cells, Tfh cells, plasmablasts, and GC B cells were seen in both GF+Tac and Tac mice compared to GF+CR or CR mice, respectively (Figures 2C–2F). In addition, the measurement of circulating IgG titers to $MSP1_{19}$ (Figures 2G–2K) and whole parasite lysate showed increased titers in Tac and GF+Tac mice compared to GF+CR and CR mice (Figure 2L). Due to similar immune responses between both Tac or CR mice and GF mice colonized with either Tac or CR cecal contents, this experiment provides proof of concept that differences in the humoral immune response are driven by gut microbiota rather than potential genetic drift between venders. These data demonstrate that differences in the composition of the gut microbiota cause differential GC responses that correlate with decreased parasite burden.

Early Abolishment of GC Structure Seen in the Spleens of Susceptible Mice Affects T:B Cell Interactions and Function

To visually evaluate the GC reactions in hyperparasitemia-resistant mice compared to hyperparasitemia-susceptible mice, spleens were evaluated by confocal microscopy. Distinct B and T cell zones were evident and spleens between Tac and CR mice were indistinguishable from one another in both naive mice and mice that were day 7 post-*P. yoelii* infection (Figure 3A). The disruption of splenic architecture during a *Plasmodium* infection is well documented (Cadman et al., 2008; Alves et al., 2015), with spleens quickly becoming dark with hemozoin deposits and expanding to almost 10× the weight of a naive spleen. Of note, this expansion and change occurs equally in Tac and CR mice (Figure S4), suggesting that the differences in the presence of GC-like structures between Tac and CR mice starting at day 9 p.i. (Figure 3A) are not a result of differing splenic expansion. By day 11 post infection, the time point when parasitemia between resistant and susceptible mice typically diverges, distinct GC-like structures are still detected in Tac mice while not evident in CR mice (Figures 3A–3C). Differences in GC numbers are consistent with the cellular analysis of $CD3^+$ cells, $GL7^+$ cells, and $B220^+$ cells from the spleens of infected mice (Figures 3D–3F). These data suggest that resistant mice have increased numbers of GC B cells and increased serum antibody titers due to the ability to maintain GC-like structures for an increased amount of time post-*Plasmodium* infection.

We next analyzed whether the increased maintenance of GC reactions in Tac mice influences T and B cell communication and GC B cell differentiation. Because T and B cell interactions are critical for the development of GC reactions, and GC B cells receive continuous instruction from Tfh cells, leading to higher affinity antibodies (Pérez-Mazliah et al., 2017), the number of T and B cell conjugates between resistant and susceptible mice was evaluated (Figure 4A). Corroborating the findings that GC B cell numbers and GC reaction numbers are similar in Tac and CR mice through day 7 post-*P. yoelii* infection, the number of T and B cell conjugates were similar between Tac and CR mice on day 7 post-*P. yoelii* infection (Figure 4B). Starting at day 9 p.i. and thereafter, Tac mice showed an increase in T and B cell conjugates (Figure 4B), suggesting that

(B–I) Spleens were removed at days 8, 11, 14, 17, and 21. Representative fluorescence-activated cell sorting (FACS) plots and total numbers of antigen-specific CD4 cells ($CD4^+CD49d^+CD11a^+$), T follicular helper cells (Tfh) ($CD4^+CD44^+CXCR5^+BCL6^+$), plasmablasts ($CD19^+B220^+IgD^-CD138^+$), and germinal center B (GC B) cells ($CD19^+CD95^+GL7^+$) were determined. Total cell number data (means ± SEs) are cumulative results (n = 6–8 mice per time point) from 2 (day 0) or 3 (days 8, 11, 14, 17, 21) independent experiments and were analyzed by unpaired t test.

(J) Serum was collected from Tac and CR mice 14 days post-*P. yoelii* infection. Sera was serially diluted and reacted against $MSP1_{19}$-coated plates. MSP1-specific antibodies were detected by ELISA. Data (means ± SDs) are representative of 3 experiments and were analyzed by unpaired t test.

*p < 0.05, **p < 0.01, ***p < 0.001, and ****p < 0.0001.

CellPress
OPEN ACCESS

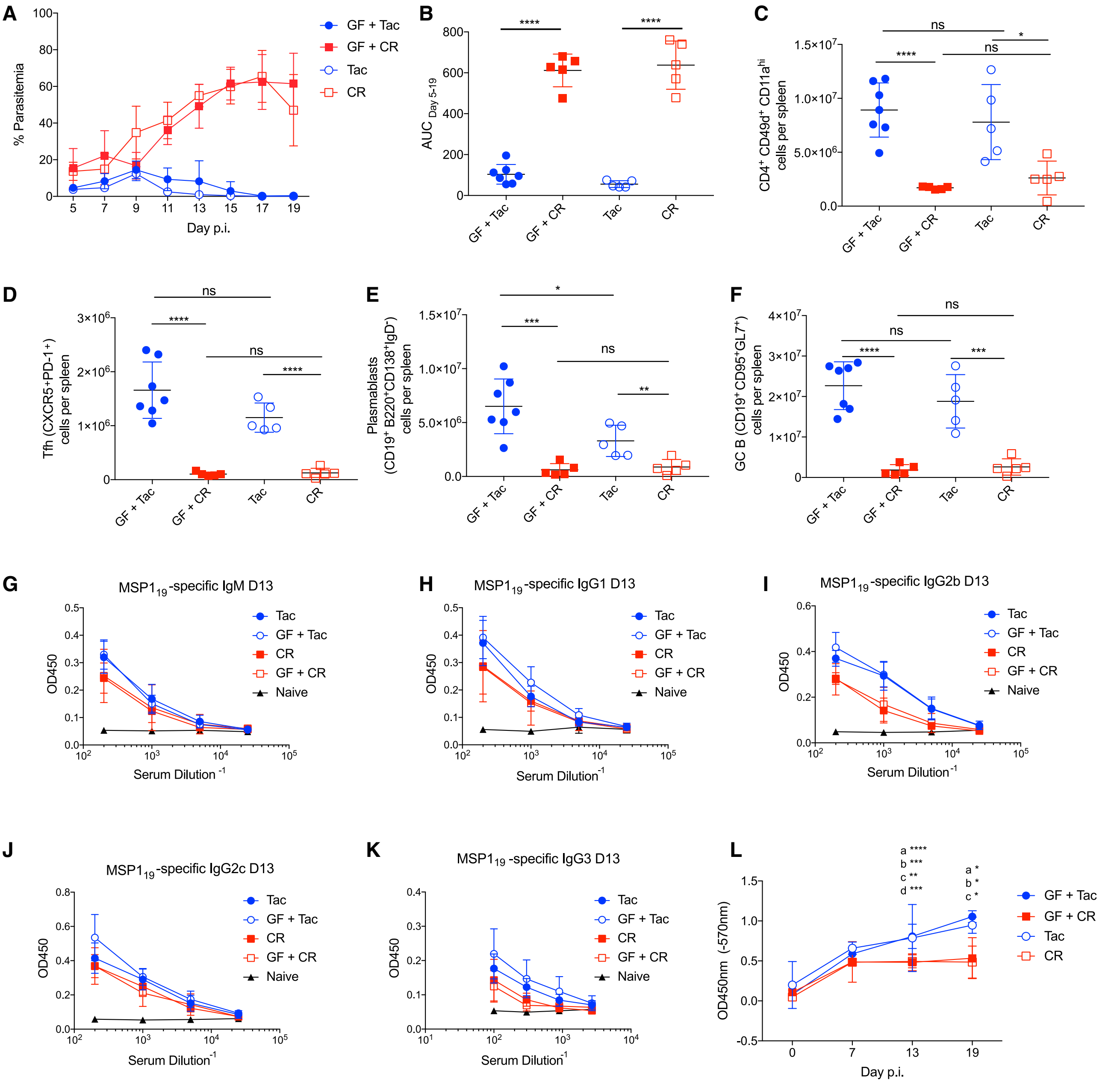

Figure 2. Gut Microbiota Composition Affects Expansion of GC B Cells and Titers of Parasite-Specific Antibodies

Germ-free mice were colonized with cecal contents from Tac (resistant) (GF-Tac; n = 7) or CR (susceptible) (GF-CR; n = 5) mice. Colonized GF mice and control Tac and CR mice (n = 5 each) were infected with *P. yoelii*.

(A) Percentage of parasitemia following *P. yoelii* infection.

(B) Area under the curve (AUC) analysis of parasitemia curves.

(C–F) Spleens were removed at day 19 and total numbers of antigen-specific CD4 cells ($CD4^+CD49^+CD11a^+$), T follicular helper cells (Tfh) ($CD4^+CD44^+CXCR5^+PD1^+$), plasmablasts ($CD19^+B220^+IgD^-CD138^+$), and GC B cells ($CD19^+CD95^+GL7^+$) were determined. Data (means ± SDs) were analyzed by unpaired t tests. *$p < 0.05$, **$p \leq 0.01$, ***$p \leq 0.001$, and ****$p \leq 0.0001$.

(G–K) Sera was collected from *P. yoelii*-naive mice or the indicated mice at day 13 post-*P. yoelii* infection and reacted against $MSP1_{19}$-coated plates to detect antibodies by ELISA.

(L) Sera from Tac, GF+Tac, CR, and GF+CR mice was collected at days 0, 7, 13, and 19 p.i., diluted at 1:200, and reacted against *P. yoelii* whole-parasite lysate-coated plates. Anti-IgG antibody was used to detect *P. yoelii*-specific IgG. Data were acquired from a single experiment and were analyzed by 2-way ANOVA. a = GF+Tac versus CR, b = GF+Tac versus GF+CR, c = Tac versus GF+CR, and d = Tac versus CR.
*$p < 0.05$, **$p \leq 0.01$, ***$p \leq 0.001$, and ****$p \leq 0.0001$.

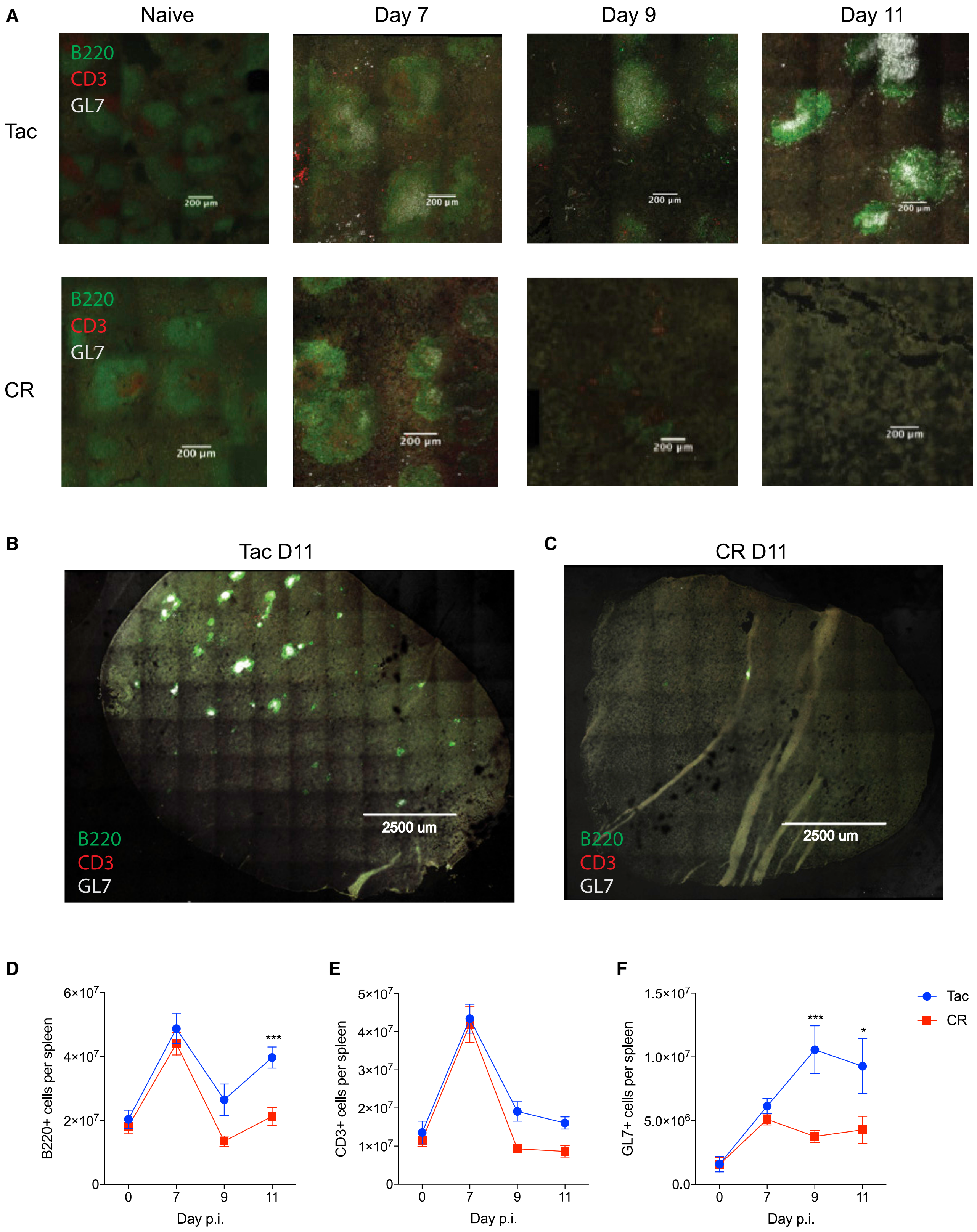

(legend on next page)

the increased ability of Tac mice to maintain GC-like structures in their spleens supports T and B cell interactions and GC B cells to thus receive necessary activation and differentiation signals. Analysis of Tfh and GC B cells in *P. yoelii*-infected Tac and CR mice showed similar expression of a range of co-stimulatory receptors on Tfh and GC B cells and the transcription factors Bcl-6 and Tcf1 in Tfh cells from Tac and CR mice (Figure S5). Expression of Bcl-6 in GC B cells, a protein necessary for the differentiation and function of GC B cells (Dent et al., 1997), is dependent on $CD4^+$ T cell co-stimulatory signals and cytokines (Nojima et al., 2011). Consistent with the increased number of T and B cell conjugates in Tac mice, there were more Bcl-6^+ GC B cells in Tac mice compared to CR mice (Figures 4C–4E).

Expression of the cell surface markers CD73 and CD80 on MBCs is associated with T cell interaction (Tomayko et al., 2010), and expression of these markers is also associated with the expression of activation-induced cytidine deaminase (AID) and the degree of somatic hypermutation (Taylor et al., 2012). Consistent with the increased numbers of T and B cell conjugates in Tac mice, an increased frequency and number of $CD73^+$ GC B cells was seen in the spleens of Tac mice compared to CR mice as the infection progressed (Figures 4F–4I), and the expression of CD73 on these GC B cells was higher in Tac mice (Figure 4I). Finally, there was an increase in the number of MBCs ($CD19^+IgD^-CD138^-CD38^+$) (Krishnamurty et al., 2016; Ryg-Cornejo et al., 2016) in Tac mice compared to CR mice (Figures 4J and 4K). Of note, when splenic cell numbers were evaluated following the clearance of *P. yoelii,* this increase in the number of MBCs in Tac mice was maintained through at least day 60 p.i. (Figure S6). In addition, following the resolution of infection, similar numbers of GC B cells, plasmablasts, and Tfh cells were quantified in spleens from Tac and CR mice, correlating with the ability of CR mice to eventually clear infection (Figure S6). Still, these cell numbers in CR mice do not reach the numbers seen in Tac mice at approximately day 19/21, providing support that despite the ability to eventually clear infection, CR do not mount as large or efficient a GC response as Tac mice. These data illustrate the increased capacity of hyperparasitemia-resistant Tac mice to maintain GC-like structures for a longer period of time following *P. yoelii* infection, which influences the interactions between B and T cells, total number of GC B cells, and output of MBCs. The CD73 expression data also suggest a potential impact on the quality of the B cell response between Tac and CR mice following *P. yoelii* infection.

Differential Signatures in the B Cell Receptor (BCR) Repertoire Associated with Gut Microbiota-Driven *Plasmodium* Resistance

To further assess potential qualitative differences in the humoral immune response between Tac and CR mice, BCR repertoire sequencing was performed on naive B cells (immunoglobulin M [IgM]) and GC B cells (IgM and IgG) isolated from spleens day 10 post-*P. yoelii* infection (Table S1). Day 10 was selected because the antibody repertoire at this time point likely captures any differences that are relevant to the ability of Tac mice to quickly clear infection in the ensuing days, while parasitemia in CR mice continues to increase (Figures 1A and 5A). Within each cell compartment and isotype, we assessed global measures of clonal abundance (Figures S7A and S7B) and repertoire diversity (Figures 5B, S7A, and S7B), as well as other repertoire features, including gene usage, complementarity determining region 3 (CDR3) length, and somatic hypermutation (SMH; Figures S7C and S7D). We observed evidence of modest clonal expansions in each group of animals among the isotypes and cell subsets (Figures S7A and S7B). While there was a trend for lower overall repertoire diversity within CR mice (Figure 5B), the observed differences between groups were not statistically significant ($p < 0.05$). No notable differences between groups were noted for CDR3 length or SHM profiles (Figures S7C and S7D). In addition, despite suggestive differences in repertoire diversity between Tac and CR groups, there was little evidence that repertoires of CR mice were dominated by biased usage of the same gene segments. Specifically, we assessed differential immunoglobulin heavy chain variable (IGHV) and IGH joining (IGHJ) gene usage between CR and Tac mice within each cell subset/isotype; no statistically significant differences were identified (Table S2; $q < 0.05$). However, we did find evidence that overall, gene usage patterns were more similar within Tac mice. Based on the repertoire dissimilarity index (RDI) (Bolen et al., 2017), we observed a greater within-group dissimilarity among IGHV segment repertoires of CR mice in the GC B cell subsets and isotypes (Figure 5C) compared to repertoires of Tac mice; this distinction was most prominent in IgG^+ GC B cells (Figure 5C). This finding was also supported using principal-component analysis of IGHV gene usage profiles among samples. Again, particularly within GC B cells, Tac mice clustered more closely as a group than CR mice (Figure S7E).

These data support the potential for Tac mice to have more diverse but consistently similar repertoires, represented by antibodies capable of targeting a greater range of *P. yoelii* antigens. This would suggest that consistency in the antibody responses within Tac mice would be associated with the production of antibodies that typically target the same/similar antigens, while the antibodies produced by CR mice target antigens that are dissimilar from their cage mates. To test this possibility, serum from individual Tac and CR mice collected 13 days post-infection was immunoblotted against *P. yoelii*-infected whole RBC lysate (Figure 5D). In support of the BCR repertoire sequencing data, Tac mice (rows 1–4) have parasite-specific antibodies binding to a much wider range of parasite antigens with greater intensity

Figure 3. Tac Mice Have Sustained Splenic GC Architecture Compared to CR Mice during *P. yoelii* Infection

Mice from Tac and CR were infected with *P. yoelii* pRBCs. Spleens from Tac and CR mice were removed at days 0, 7, 9, and 11 p.i., frozen, sectioned, and mounted on slides. Tissue sections were stained for anti-B220 (green), anti-CD3 (red), and anti-GL7 (gray), and analyzed through confocal microscopy.

(A–C) Representative spleen sections of Tac and CR mice on the indicated day (A). Stitched mosaic image of Tac spleen D11 (B) and CR spleen D11 p.i. (C).

(D–F) Total $B220^+$ (D), (E) $CD3^+$, and (F) $GL7^+$ cells per spleens of infected mice at days 0, 7, 9, and 11. Data (means ± SEs) are cumulative results from 2–3 independent experiments per time point and were analyzed by 2-way ANOVA; multiple comparisons were analyzed with Tukey's post hoc multiple comparisons test; $^*p < 0.05$, and $^{***}p < 0.001$.

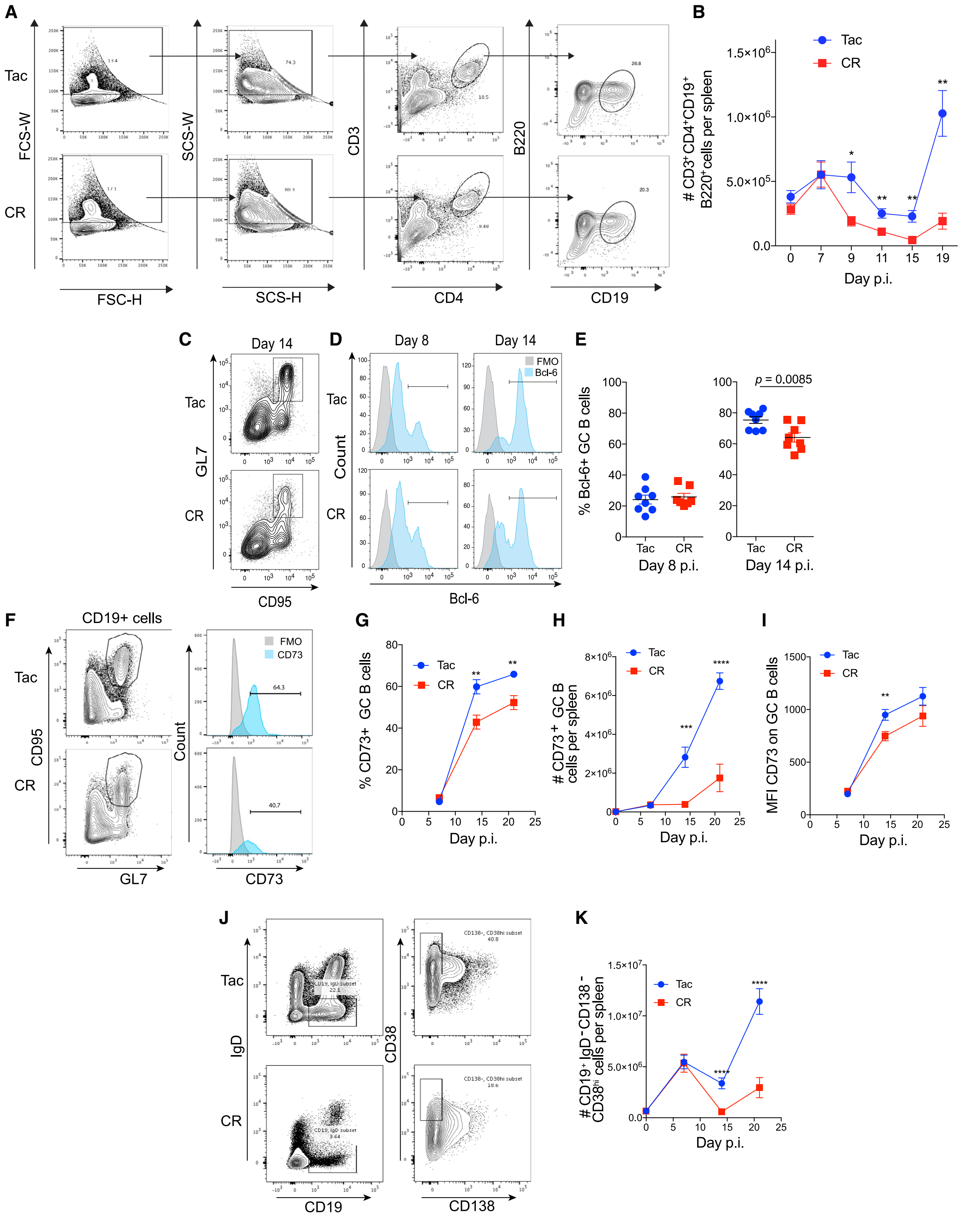

(legend on next page)

compared to CR mice (rows 5–8). In addition to having antibodies that bind to a greater number of antigens, Tac mice also show more consistent responses (i.e., binding to many of the same antigens) between one another that are not seen in CR mice (e.g., bands noted by arrows 3, 4, and 5). Importantly, there are key differences in the banding pattern that suggest that differences in the western blot between Tac and CR serum are not driven by an increase in antibody quantity in Tac mice compared to CR mice. For example, although a band indicated by arrow 4 is detected in all 4 CR mice, CR lane 1, which exhibits a weaker signal than lanes 2 and 4, has a detectable band (arrow 5) that is not detected in CR lanes 2–4. In addition, band 4 in CR lane 2 exhibits an intensity similar to that seen in Tac lanes 2–4, but CR lane 2 does not exhibit bands at arrows 1, 2, and 5–7 that are seen in Tac lanes. Thus, independent of antibody abundance, the data suggest that CR mice do not consistently target similar *P. yoelii* antigens, compared to the more consistent banding pattern observed with Tac mice, and CR mice do not appear to target the full diversity of *P. yoelii* antigens that elicit antibody responses in Tac mice. In sum, these data suggest that due to the increased longevity of GC reactions and greater T cell and B cell interactions in Tac mice, GC B cells from Tac mice have a more targeted, informed response, leading to an increased range of IGHV segments used and an antibody response more efficient at eliminating *P. yoelii.*

Gut Microbiota Function through GC Reactions to Provide Protection against *P. yoelii* Hyperparasitemia

Collectively, the data led to the hypothesis that gut microbiota regulate GC reactions to control parasite burden. To test this hypothesis, *P. yoelii*-infected Tac and CR mice were treated with MR1, a monoclonal antibody that blocks CD40L, to prevent T and B cell interactions and the formation of GC reactions (Figure 6A) (Elgueta et al., 2009). If the gut microbiota shapes the severity of malaria independent of GC reactions, then disrupting GCs would have a minimal effect on parasite burden. Following MR1 treatment, there were relatively minimal effects on $CD4^+$ T cell responses compared to B cell responses, in which GC B cell differentiation was blocked in both Tac and CR mice (Figures 6B and 6C). Consistent with the block in GC B cell differentiation, $MSP1_{19}$-specific antibodies did not undergo isotype switching in MR1-treated mice (Figure 6D). In support of the hypothesis, both Tac and CR mice treated with MR1 showed overlapping parasitemia kinetics and total parasite burdens that were similar to that seen in control CR mice (Figures 6E and 6F). These data identify the modulation of GC reactions as a mechanism by which gut microbiota affects the severity of malaria.

Resistance to *P. yoelii* Hyperparasitemia Results in Protection against Lethal *P. berghei* Infection

Individuals living in malaria-endemic regions must be exposed to *Plasmodium* repeatedly over multiple years to develop an antibody threshold that confers resistance to severe malaria. Intriguingly, Tac mice had increased numbers of MBCs following *P. yoelii* infection compared to CR mice (Figures 4J and 4K), and these numbers were sustained through at least day 60 p.i. (Figure S6). Therefore, to determine whether gut microbiota-dependent resistance to *P. yoelii* seen in Tac mice led to resistance to subsequent *Plasmodium* infection, mice were challenged with lethal *P. berghei* ANKA 60 days after initial *P. yoelii* challenge (Figure 7A). Mice exhibited expected parasite kinetics following *P. yoelii* infection (Figure 7B), with CR mice having increased parasitemia compared to Tac mice (Figure 7C). *P. berghei* ANKA is typically a lethal infection in C57BL/6 mice, with the majority of mice developing experimental cerebral malaria (ECM) by day 10 p.i., and any surviving mice succumbing to hyperparasitemia soon after (Bagot et al., 2002). Control, *P. yoelii*-naive Tac, and CR mice exhibited 100% mortality from ECM following *P. berghei* ANKA infection (Figure 7D). However, significantly (Gehan-Breslow-Wilcoxon test; $p = 0.0002$) more *P. yoelii*-immune Tac mice (86%) survived ECM following challenge with *P. berghei,* compared to *P. yoelii*-immune CR mice (30%) (Figure 7D). Approximately 60% of *P. yoelii*-immune Tac mice that survived ECM were also able to prevent the development of *P. berghei* hyperparasitemia and control the infection (Figure 7E). In contrast, none of the *P. yoelii*-immune CR mice that survived ECM were able to control *P. berghei* parasitemia (Figure 7E). In addition, *P. yoelii*-immune Tac mice that developed hyperparasitemia showed decreases in parasitemia and survived longer than *P. yoelii*-immune CR mice that developed hyperparasitemia (Figures 7E and 7F). These data show that gut microbiota-induced resistance to *P. yoelii* hyperparasitemia allows for better survival following lethal *P. berghei* challenge.

Figure 4. Resistant Mice Exhibit Increased T:B Cell Interactions and B Cell Functionality

Resistant and susceptible mice were infected with *P. yoelii* pRBCs.

(A) Representative contour plots and flow cytometry gating of splenocytes at day 9 p.i. to identify T and B cell conjugates.

(B) Total number of T and B cell conjugates ($CD3^+CD4^+CD19^+B220^+$ doublets) per spleen on the indicated day p.i. Data (means ± SEs) are from 2 independent experiments (n = 8 mice per time point, day 9, n = 4) were analyzed by unpaired t test.

(C) Representative contour plots (gated on $B220^+CD19^+$ cells) on day 14 p.i. to identify GC B cells ($GL7^+CD95^+$).

(D) Representative histograms to identify $BCL\text{-}6^+$ GC B cells.

(E) Percentage of $BCL\text{-}6^+$ GC B cells per spleen at days 8 and 14 p.i. Data (means ± SEs) from 3 independent experiments (n = 8 mice per time point) were analyzed by unpaired t test.

(F) Representative flow cytometry graphs identifying $CD73^+$ GC B cells from spleens at day 21 p.i.

(G) Percentage of $CD73^+$ GC B cells per spleen.

(H) Total number of $CD73^+$ GC B cells per spleen.

(I) Mean fluorescence intensity (MFI) of CD73 on GC B cells.

(J) Representative contour plots for $CD19^+IgD^-CD138^-CD38^{hi}$ memory B cells (MBCs).

(K) Total number of MBCs per spleen on the indicated day p.i. Data (means ± SEs) from 2 independent experiments (n = 10 mice per time point) were analyzed by unpaired t test.

(G–I) Data (means ± SEs) from 2 independent experiments (n = 10 mice per time point) were analyzed by unpaired t tests.

*$p < 0.05$, **$p \leq 0.01$, ***$p \leq 0.001$, and ****$p \leq 0.0001$.

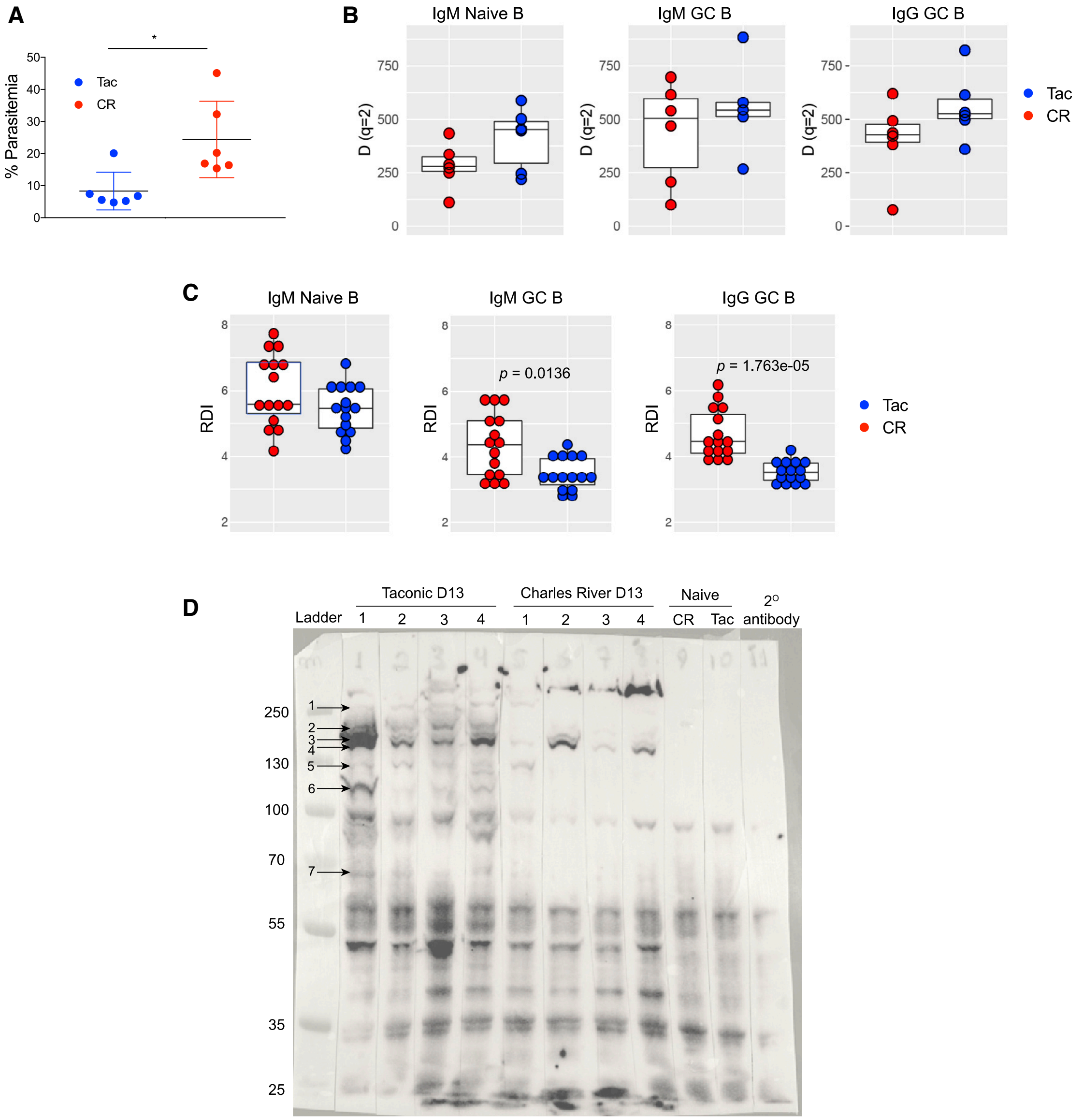

Figure 5. Qualitative Differences in BCR Repertoire in Hyperparasitemia-Resistant and -Susceptible Mice

Spleens were removed from Tac and CR mice (n = 6) at day 10 p.i. Naive B cells ($CD19^{+}CD95^{-}GL7^{-}IgD^{+}IgM^{+}$) and GC B cells ($CD19^{+}CD95^{+}GL7^{+}$) were sorted from each spleen, RNA was extracted, and IgM and IgG libraries were generated.

(A) Parasitemia of mice before spleen removal.

(B) Repertoire diversity at diversity order, q = 2, in naive cell, IgM^{+} GC B cells, and IgG^{+} GC B cells.

(C) Repertoire dissimilarity index (RDI), based on overall repertoire IGHV gene usage profiles in naive cells, IgM^{+} GC B cells, and IgG^{+} GC B cells. Between-group comparisons were made using the Kruskal-Wallace test.

(D) Western blot from D13 serum (serum collected from Figure 2 control mice) against *P. yoelii* whole-parasite lysate.

DISCUSSION

While previous studies have illustrated that the absence of gut microbiota negatively affects humoral immunity (Ichinohe et al., 2011; Abt et al., 2012), this study uniquely demonstrates that the composition of gut microbiota influences the overall magnitude and quality of GC reactions and thus the severity of an infectious disease, including memory development and protection from re-infection. These data show that differences in the gut microbiota in otherwise genetically identical mice lead to differential GC reaction persistence at critical immune time points between days 9 and 14 post-*P. yoelii* infection, when a divergence in parasitemia becomes evident. This differential maintenance in GC structures results in different numbers of GC-relevant cell types, differential *Plasmodium*-specific antibody titers, and differences in the quality and specificity of these antibodies to the *Plasmodium* parasite. Upon a second *Plasmodium* challenge, these differences in the initial GC response in resistant mice

CellPress
OPEN ACCESS

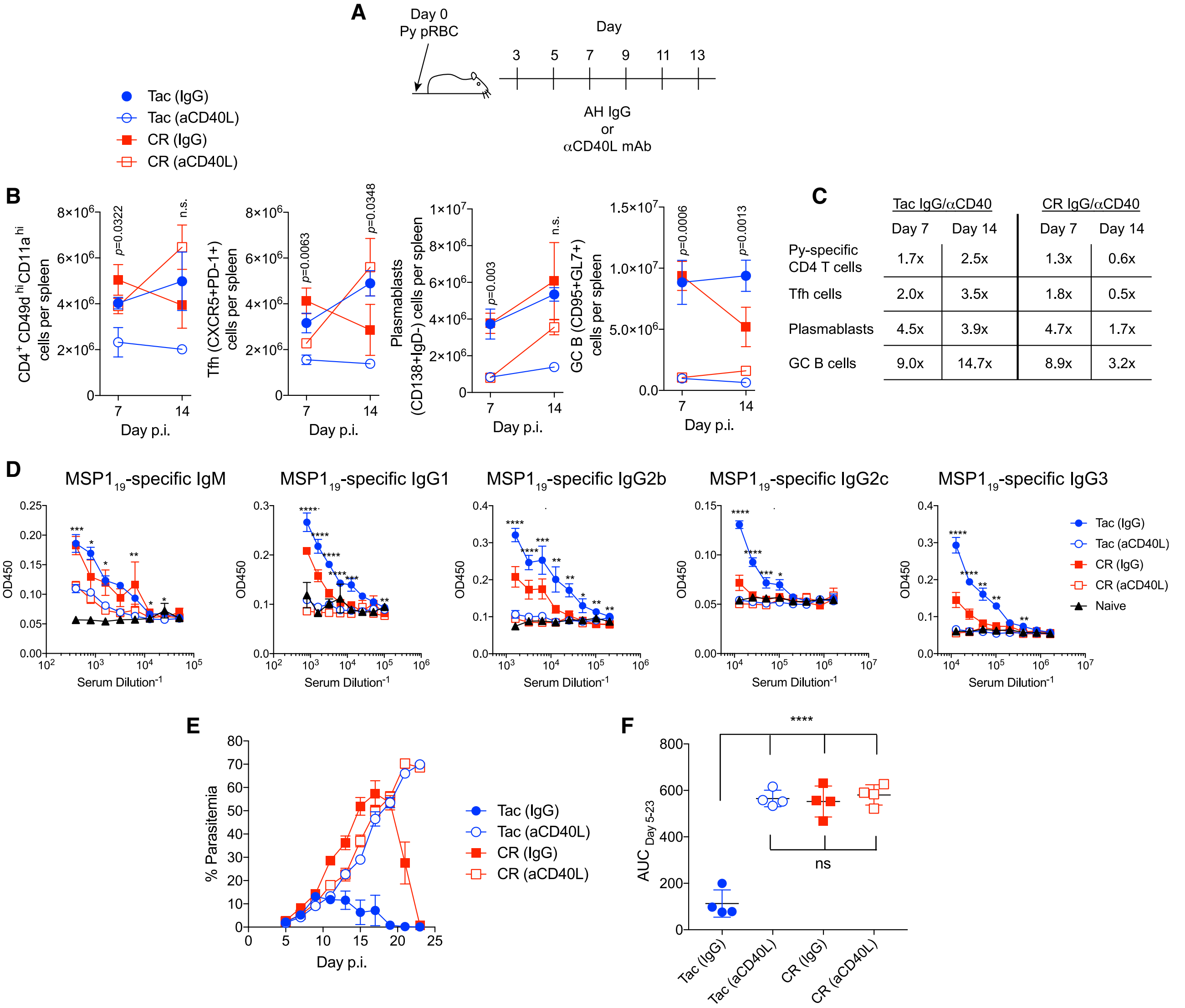

	Tac IgG/αCD40		CR IgG/αCD40	
	Day 7	Day 14	Day 7	Day 14
Py-specific CD4 T cells	1.7x	2.5x	1.3x	0.6x
Tfh cells	2.0x	3.5x	1.8x	0.5x
Plasmablasts	4.5x	3.9x	4.7x	1.7x
GC B cells	9.0x	14.7x	8.9x	3.2x

Figure 6. Gut Microbiota Modulates GC Reactions to Control *P. yoelii* Infections

(A) Tac and CR mice were infected with *P. yoelii* and treated with either αCD40L or IgG (control) on days 3, 5, 7, 9, 11, and 13.

(B) Total numbers per spleen of antigen-specific $CD4^+$ cells, Tfh cells, plasmablasts, and GCB cells on days 7 and 14 p.i. Data (means ± SEs) from 3 mice/group are representative results from 2 experiments and were analyzed by 1-way ANOVA and Tukey's multiple comparison post-test.

(C) Ratio of cell numbers from (B) of IgG-treated mice versus αCD40L-treated mice at days 7 and 14 p.i.

(D) $MSP1_{19}$-specific antibody titers on day 21 p.i. Data (means ± SEs) from 3–4 mice/group are representative results from 2 experiments and were analyzed by 1-way ANOVA.

(E) Percentage of parasitemia following *P. yoelii* infection. Data (means ± SEs) from 4 mice/group are representative data from 2 experiments.

(F) AUC analysis. Data (means ± SDs) from 4 mice/group are representative data from 2 experiments and were analyzed by 1-way ANOVA and Tukey's multiple comparison post-test.

n.s., not significant, *p < 0.05, **p < 0.01, ***p < 0.001, ****p < 0.0001.

led to an increased ability to prevent the development of symptoms of ECM and hyperparasitemia. While a previous study by Teng et al. (2016) has shown the influence of gut microbiota on Tfh cells in gut-distal sites in an arthritis model, these Tfh cells were primed and differentiated in Peyer's patches before systemic migration. Our study is unique in that, based on our analysis, gut microbiota composition affects spleen GC B cell biology, rather than Tfh cell biology, to influence the severity of *Plasmodium* infection. Moreover, these GC B cells differentiate in the spleen rather than in gut-associated tissues, as was observed in the previous study. While the majority of these experiments were done in the context of *Plasmodium* infections, the differences seen in GC B numbers between mice from different vendors immunized with sheep RBCs suggest that these gut microbiota-mediated differences in the GC response are not limited to malaria. These findings have implications for

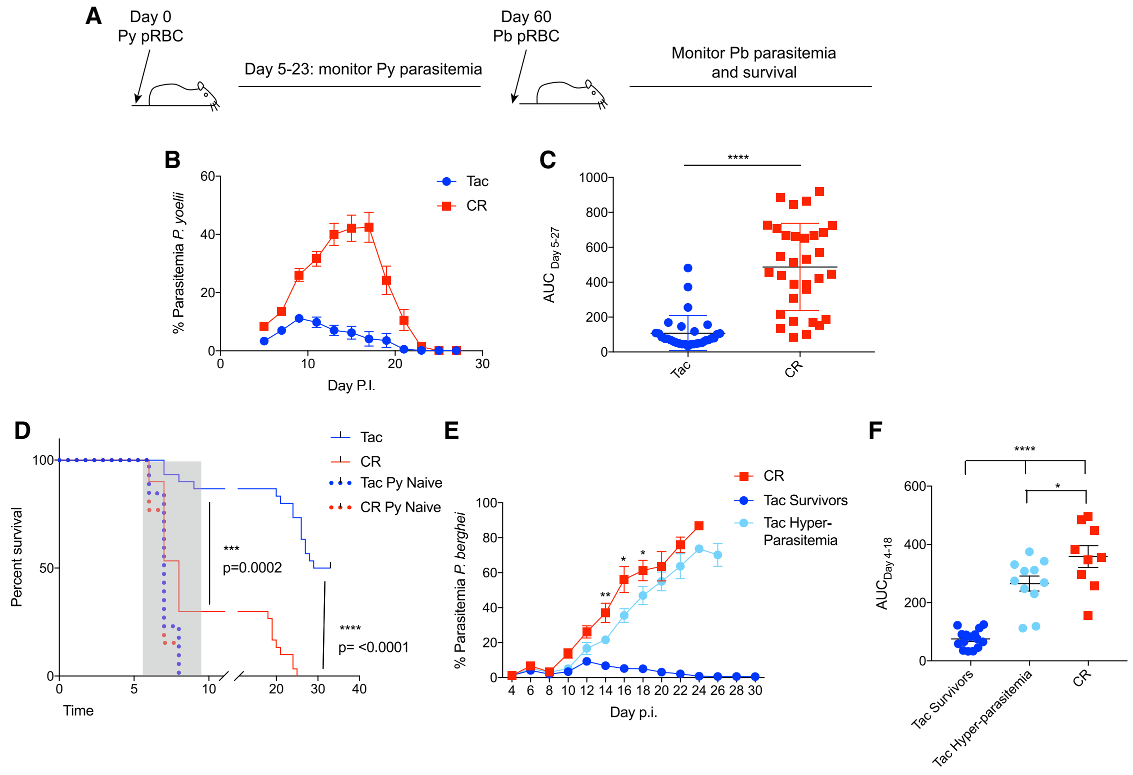

Figure 7. *P. yoelii*-Resistant Mice Exhibit Increased Protection to Lethal *P. berghei* Challenge

(A) Tac and CR mice were infected with *P. yoelii* pRBCs. Sixty days post-*P. yoelii* infection, mice were infected with *P. berghei* ANKA pRBCs.

(B) Percentage of parasitemia of *P. yoelii* infection.

(C) AUC analysis of *P. yoelii* parasitemia. Data (means ± SEs) were collected from 4 independent experiments (n = 30–33 mice/group) and analyzed by unpaired t test.

(D) Survival curve of mice following *P. berghei* ANKA infection. Deaths associated with experimental cerebral malaria (ECM) occurred between days 5 and 10 p.i. and are shaded in gray. Data (means ± SEs) were collected from 4 independent experiments (n = 30–33 Tac and CR; n = 13 Py Naive mice) and analyzed by Gehan-Breslow-Wilcoxon test.

(E) Percentage of parasitemia of *P. berghei* ANKA infection. Statistical significance indicated on parasitemia curve shows significance in parasitemia between CR mice and Tac hyperparasitemia mice at individual days p.i. and was determined by unpaired t test.

(F) AUC analysis of *P. berghei* ANKA parasitemia (only mice that did not develop ECM are shown). Data (means ± SEs) were analyzed by 1-way ANOVA and Tukey's multiple comparison post-test. *$p < 0.05$ and ****$p < 0.0001$.

potential treatments in a wide range of antibody-mediated and antibody-dependent diseases and offer the scientific community increased perspectives on the importance of an individual's gut microbiota composition.

One of the challenges researchers have faced in the production of an effective and long-lasting vaccine to *Plasmodium* is the large number of surface antigens present on the parasite and infected RBCs (Le Roch et al., 2004). Several factors contribute to this antigen diversity, including the complex life cycle of *Plasmodium* and mechanisms of immune evasion, which results in the need for individuals to be exposed to repeated infections over a period of years to develop naturally acquired immunity to malaria (Ferreira et al., 2004). Naturally acquired immunity has been shown to be attributed to higher titers of *Plasmodium*-specific antibodies and, potentially more important, an increase in the number of the antigens that these antibodies recognize (Osier et al., 2008, Nogaro et al., 2011). Our data show that the BCR repertoires of susceptible mice had lower diversity overall, particularly in the GC B cell compartment, characterized by more restricted yet larger clonal expansion. However, despite the increased diversity observed in resistant mice, their GC B cell repertoires looked more similar between one another than those of the susceptible mice. These data suggested that susceptible mice potentially have a more limited breadth of circulating *Plasmodium* antigen-specific antibodies, an idea that was corroborated through western blot. The BCR repertoire analysis of resistant and susceptible mice in combination with the evidence of decreased longevity of visual GC reactions in susceptible mice through confocal microscopy suggest that lack of GC structure maintenance renders the GC B cells in these mice unable to develop the requisite antigen-specific antibody repertoire necessary for early parasite control.

Although this study was performed using a mouse model, several recent human studies have also begun to identify the composition of the gut microbiota as a factor in *Plasmodium* risk and severity in human populations (Yooseph et al., 2015; Mandal et al., 2019). Further research has shown that the presence of specific human commensal organisms can contribute

to the production of antibodies that are cross-protective against *Plasmodium* infection (Yilmaz et al., 2014). Whether the human gut microbiota also contributes to the breadth and magnitude of *P. falciparum*-specific antibodies or GC reaction development, however, remains unknown. Nevertheless, these data point to the possibility of gut microbiota modulation to elicit the robust and enduring humoral immune response that is critical for a malaria vaccine to be effective. Therefore, understanding the role of individuals' gut microbiota on their ability to produce an antibody response is relevant to the continued efforts to produce an efficient, long-lasting vaccine to *Plasmodium*. Furthermore, acknowledging the role of the gut microbiota in the humoral immune response may become important in determining the best way for a vaccine or treatment to be effective to the greatest percentage of a population for a wide range of infections and diseases.

One of the most striking observations in this study was the ability of 50% of *P. yoelii*-resistant mice to survive a lethal *P. berghei* challenge. While recognizing the limitations of the murine model of malaria, these data demonstrate the importance that efficient GC responses during an initial *Plasmodium* infection, not only toward control of that infection but also subsequent infections, could have on preventing severe malaria and thus morbidity and mortality in human populations. Because individuals in endemic areas require repeated exposures to develop a protective antibody threshold, better understanding of the role of the gut microbiota in this response provides fundamental new knowledge regarding the generation of anti-*Plasmodium* antibody responses. Furthermore, this will afford the possibility for the rational development of approaches aimed at manipulating the microbiota to increase the rate by which young children or previously naive individuals can develop this protective threshold.

Although this study links the composition of the gut microbiota to the quality of the GC response, future work is required to better determine the details of the mechanism linking the gut microbiota to the humoral immune response. Prior studies have linked gut microbiota-produced short-chain fatty acids to B cell responses (Kamada et al., 2013); however, we observed no correlation with short-chain fatty acid levels and the severity of malaria (Chakravarty et al., 2019). Although our lab has previously looked at sequencing data to determine the differences in the microbial composition between resistant and susceptible mice (Villarino et al., 2016), a specific microbe, group of microbes, or microbial product driving the differences in infection severity and corresponding immune responses has yet to be identified. In addition to the challenges in identifying the microbial factor leading to the differences in humoral immunity following *P. yoelii* infection, it is possible that the mechanism leading to these differences is slight or localized to a specific cell type due to the intricacies of the immune response to *Plasmodium* infection and interactions with gut microbiota. This leaves many experiments still to be done to link specific compositions and functions of gut microbiota to differences in GC reactions following *Plasmodium* infection.

In conclusion, these data show that gut microbiota contributes toward differences in the susceptibility to *Plasmodium* infection in mice through modulating the magnitude and quality of spleen GC reactions. Moreover, these differences lead to long-term resistance to subsequent *Plasmodium* infection. Although the molecular mechanism linking the gut microbiota and the humoral immune response has yet to be determined, the present study supports the potential of gut microbiota modulation to increase the rate of *Plasmodium*-specific acquired immunity, thus lessening disease severity and malaria-associated mortality in susceptible populations.

STAR★METHODS

Detailed methods are provided in the online version of this paper and include the following:

- KEY RESOURCES TABLE
- RESOURCE AVAILABILITY
  - Lead Contact
  - Materials Availability
  - Data and Code Availability
- EXPERIMENTAL MODEL AND SUBJECT DETAILS
  - Mice
  - Plasmodium Infections
- METHOD DETAILS
  - Evaluation of parasitemia
  - Cellular immune response
  - Antibodies
  - Confocal Microscopy
  - ELISA
  - Expressed Antibody Repertoire Sequencing and Analysis
  - BCR Rep Sequencing Processing and Analysis
  - Western Blot
  - Antibody treatments
  - Evaluation of Iron Levels
  - Phagocytosis Assay
  - Sheep Red Blood Cell Injections
- QUANTIFICATION AND STATISTICAL ANALYSIS

SUPPLEMENTAL INFORMATION

Supplemental Information can be found online at https://doi.org/10.1016/j.celrep.2020.108503.

ACKNOWLEDGMENTS

The authors would like to thank Drs. Mark Kaplan, Martin Richer, and Tuan Tran for critical review of the manuscript. This work was supported in part by grants from the National Institute of Allergy and Infectious Disease of the National Institutes of Health (1R21AI113386 and 1R01AI123486) to N.W.S. The content is solely the responsibility of the authors and does not necessarily represent the official views of the National Institutes of Health. The project was supported by the Indiana University Health – Indiana University School of Medicine Strategic Research Initiative to N.W.S. Support provided by the Herman B. Wells Center to N.W.S. came in part from the Riley Children's Foundation. The authors also thank the members of the Indiana University Melvin and Bren Simon Cancer Center Flow Cytometry Resource Facility (FCRF) for their outstanding technical support. The FCRF is funded in part by National Cancer Institute grant P30 CA082709, National Institute of Diabetes and Digestive and Kidney Diseases (NIDDK) grant U54 DK106846, and National Institutes of Health instrumentation grant 1S10D012270.

AUTHOR CONTRIBUTIONS

Conceptualization, M.L.W. and N.W.S.; Formal Analysis, J.K., D.A.T., and C.T.W.; Investigation, M.L.W., R.P., W.L.P., J.E.D., J.K., and N.W.S.; Writing – Original Draft, M.L.W. and N.W.S.; Writing – Review & Editing, M.L.W., R.P., C.T.W., and N.W.S.; Visualization, M.L.W., R.P., J.K., D.A.T., C.T.W., and N.W.S.; Supervision, C.T.W. and N.W.S.; Funding Acquisition, N.W.S.

DECLARATION OF INTERESTS

The authors declare no competing interests.

Received: June 13, 2020
Revised: October 9, 2020
Accepted: November 18, 2020
Published: December 15, 2020

REFERENCES

Abt, M.C., Osborne, L.C., Monticelli, L.A., Doering, T.A., Alenghat, T., Sonnenberg, G.F., Paley, M.A., Antenus, M., Williams, K.L., Erikson, J., et al. (2012). Commensal bacteria calibrate the activation threshold of innate antiviral immunity. Immunity *37*, 158–170.

Alves, F.A., Pelajo-Machado, M., Totino, P.R., Souza, M.T., Gonçalves, E.C., Schneider, M.P., Muniz, J.A., Krieger, M.A., Andrade, M.C., Daniel-Ribeiro, C.T., and Carvalho, L.J. (2015). Splenic architecture disruption and parasite-induced splenocyte activation and anergy in Plasmodium falciparum-infected Saimiri sciureus monkeys. Malar. J. *14*, 128.

Arpaia, N., Campbell, C., Fan, X., Dikiy, S., van der Veeken, J., deRoos, P., Liu, H., Cross, J.R., Pfeffer, K., Coffer, P.J., and Rudensky, A.Y. (2013). Metabolites produced by commensal bacteria promote peripheral regulatory T-cell generation. Nature *504*, 451–455.

Ashley, E.A., Dhorda, M., Fairhurst, R.M., Amaratunga, C., Lim, P., Suon, S., Sreng, S., Anderson, J.M., Mao, S., Sam, B., et al.; Tracking Resistance to Artemisinin Collaboration (TRAC) (2014). Spread of artemisinin resistance in Plasmodium falciparum malaria. N. Engl. J. Med. *371*, 411–423.

Bagot, S., Idrissa Boubou, M., Campino, S., Behrschmidt, C., Gorgette, O., Guénet, J.L., Penha-Gonçalves, C., Mazier, D., Pied, S., and Cazenave, P.A. (2002). Susceptibility to experimental cerebral malaria induced by Plasmodium berghei ANKA in inbred mouse strains recently derived from wild stock. Infect. Immun. *70*, 2049–2056.

Benjamini, Y., and Hochberg, Y. (1995). Controlling the false discovery rate: a practical and powerful approach to multiple testing. J. R. Stat. Soc. B *57*, 289–300.

Blackman, M.J., Heidrich, H.G., Donachie, S., McBride, J.S., and Holder, A.A. (1990). A single fragment of a malaria merozoite surface protein remains on the parasite during red cell invasion and is the target of invasion-inhibiting antibodies. J. Exp. Med. *172*, 379–382.

Bolen, C.R., Rubelt, F., Vander Heiden, J.A., and Davis, M.M. (2017). The Repertoire Dissimilarity Index as a method to compare lymphocyte receptor repertoires. BMC Bioinformatics *18*, 155.

Burel, J.G., Apte, S.H., Groves, P.L., Boyle, M.J., Langer, C., Beeson, J.G., McCarthy, J.S., and Doolan, D.L. (2017). Dichotomous miR expression and immune responses following primary blood-stage malaria. JCI Insight *2*, 93434.

Cadman, E.T., Abdallah, A.Y., Voisine, C., Sponaas, A.M., Corran, P., Lamb, T., Brown, D., Ndungu, F., and Langhorne, J. (2008). Alterations of splenic architecture in malaria are induced independently of Toll-like receptors 2, 4, and 9 or MyD88 and may affect antibody affinity. Infect. Immun. *76*, 3924–3931.

Chakravarty, S., Mandal, R.K., Duff, M.L., Yuan, F., Zhang, X., and Schmidt, N.W. (2019). Correction: intestinal short-chain fatty acid composition does not explain gut microbiota-mediated effects on malaria severity. PLOS ONE *14*, e0218250.

Chao, A., et al. (2014). Rarefaction and extrapolation with Hill numbers: a framework for sampling and estimation in species diversity studies. Ecol. Monogr. *84*, 45–67.

Chao, A., Hsieh, T.C., Chazdon, R.L., Colwell, R.K., and Gotelli, N.J. (2015). Unveiling the species-rank abundance distribution by generalizing the Good-Turing sample coverage theory. Ecology *96*, 1189–1201.

Cohen, S., McGregor, I.A., and Carrington, S. (1961). Gamma-globulin and acquired immunity to human malaria. Nature *192*, 733–737.

Crompton, P.D., Kayala, M.A., Traore, B., Kayentao, K., Ongoiba, A., Weiss, G.E., Molina, D.M., Burk, C.R., Waisberg, M., Jasinskas, A., et al. (2010). A prospective analysis of the Ab response to Plasmodium falciparum before and after a malaria season by protein microarray. Proc. Natl. Acad. Sci. USA *107*, 6958–6963.

Crotty, S. (2014). T follicular helper cell differentiation, function, and roles in disease. Immunity *41*, 529–542.

Denny, J.E., Powers, J.B., Castro, H.F., Zhang, J., Joshi-Barve, S., Campagna, S.R., and Schmidt, N.W. (2019). Differential Sensitivity to Plasmodium yoelii Infection in C57BL/6 Mice Impacts Gut-Liver Axis Homeostasis. Sci. Rep. *9*, 3472.

Dent, A.L., Shaffer, A.L., Yu, X., Allman, D., and Staudt, L.M. (1997). Control of inflammation, cytokine expression, and germinal center formation by BCL-6. Science *276*, 589–592.

Durack, J., and Lynch, S.V. (2019). The gut microbiome: relationships with disease and opportunities for therapy. J. Exp. Med. *216*, 20–40.

Elgueta, R., Benson, M.J., de Vries, V.C., Wasiuk, A., Guo, Y., and Noelle, R.J. (2009). Molecular mechanism and function of CD40/CD40L engagement in the immune system. Immunol. Rev. *229*, 152–172.

Ferreira, M.U., da Silva Nunes, M., and Wunderlich, G. (2004). Antigenic diversity and immune evasion by malaria parasites. Clin. Diagn. Lab. Immunol. *11*, 987–995.

Figueiredo, M.M., Costa, P.A.C., Diniz, S.Q., Henriques, P.M., Kano, F.S., Tada, M.S., Pereira, D.B., Soares, I.S., Martins-Filho, O.A., Jankovic, D., et al. (2017). T follicular helper cells regulate the activation of B lymphocytes and antibody production during Plasmodium vivax infection. PLOS Pathog. *13*, e1006484.

Gallup, J.L., and Sachs, J.D. (2001). The economic burden of malaria. Am. J. Trop. Med. Hyg. *64* (*1-2 Suppl*), 85–96.

Giudicelli, V., Duroux, P., Ginestoux, C., Folch, G., Jabado-Michaloud, J., Chaume, D., and Lefranc, M.P. (2006). IMGT/LIGM-DB, the IMGT comprehensive database of immunoglobulin and T cell receptor nucleotide sequences. Nucleic Acids Res. *34*, D781–D784.

Gupta, N.T., Vander Heiden, J.A., Uduman, M., Gadala-Maria, D., Yaari, G., and Kleinstein, S.H. (2015). Change-O: a toolkit for analyzing large-scale B cell immunoglobulin repertoire sequencing data. Bioinformatics *31*, 3356–3358.

Guthmiller, J.J., Graham, A.C., Zander, R.A., Pope, R.L., and Butler, N.S. (2017). Cutting Edge: IL-10 Is Essential for the Generation of Germinal Center B Cell Responses and Anti-Plasmodium Humoral Immunity. J. Immunol. *198*, 617–622.

Hamilton, W.L., Amato, R., van der Pluijm, R.W., Jacob, C.G., Quang, H.H., Thuy-Nhien, N.T., Hien, T.T., Hongvanthong, B., Chindavongsa, K., Mayxay, M., et al. (2019). Evolution and expansion of multidrug-resistant malaria in southeast Asia: a genomic epidemiology study. Lancet Infect. Dis. *19*, 943–951.

Hill, M.O. (1973). Diversity and Evenness: A Unifying Notation and Its Consequences. Ecology *54*, 427–432.

Hill, D.L., Eriksson, E.M., Li Wai Suen, C.S., Chiu, C.Y., Ryg-Cornejo, V., Robinson, L.J., Siba, P.M., Mueller, I., Hansen, D.S., and Schofield, L. (2013). Opsonising antibodies to P. falciparum merozoites associated with immunity to clinical malaria. PLOS ONE *8*, e74627.

Ichinohe, T., Pang, I.K., Kumamoto, Y., Peaper, D.R., Ho, J.H., Murray, T.S., and Iwasaki, A. (2011). Microbiota regulates immune defense against

respiratory tract influenza A virus infection. Proc. Natl. Acad. Sci. USA *108*, 5354–5359.

Kamada, N., and Núñez, G. (2014). Regulation of the immune system by the resident intestinal bacteria. Gastroenterology *146*, 1477–1488.

Kamada, N., Seo, S.U., Chen, G.Y., and Núñez, G. (2013). Role of the gut microbiota in immunity and inflammatory disease. Nat. Rev. Immunol. *13*, 321–335.

Kim, M., and Kim, C.H. (2017). Regulation of humoral immunity by gut microbial products. Gut Microbes *8*, 392–399.

Kim, M., Qie, Y., Park, J., and Kim, C.H. (2016). Gut Microbial Metabolites Fuel Host Antibody Responses. Cell Host Microbe *20*, 202–214.

Krishnamurty, A.T., Thouvenel, C.D., Portugal, S., Keitany, G.J., Kim, K.S., Holder, A., Crompton, P.D., Rawlings, D.J., and Pepper, M. (2016). Somatically Hypermutated Plasmodium-Specific IgM(+) Memory B Cells Are Rapid, Plastic, Early Responders upon Malaria Rechallenge. Immunity *45*, 402–414.

Le Roch, K.G., Johnson, J.R., Florens, L., Zhou, Y., Santrosyan, A., Grainger, M., Yan, S.F., Williamson, K.C., Holder, A.A., Carucci, D.J., et al. (2004). Global analysis of transcript and protein levels across the Plasmodium falciparum life cycle. Genome Res. *14*, 2308–2318.

Lécuyer, E., Rakotobe, S., Lengliné-Garnier, H., Lebreton, C., Picard, M., Juste, C., Fritzen, R., Eberl, G., McCoy, K.D., Macpherson, A.J., et al. (2014). Segmented filamentous bacterium uses secondary and tertiary lymphoid tissues to induce gut IgA and specific T helper 17 cell responses. Immunity *40*, 608–620.

Mandal, R.K., Crane, R.J., Berkley, J.A., Gumbi, W., Wambua, J., Ngoi, J.M., Ndungu, F.M., and Schmidt, N.W. (2019). Longitudinal Analysis of Infant Stool Bacteria Communities Before and After Acute Febrile Malaria and Artemether-Lumefantrine Treatment. J. Infect. Dis. *220*, 687–698.

Marsh, K., and Kinyanjui, S. (2006). Immune effector mechanisms in malaria. Parasite Immunol. *28*, 51–60.

McAllister, E.J., Apgar, J.R., Leung, C.R., Rickert, R.C., and Jellusova, J. (2017). New Methods To Analyze B Cell Immune Responses to Thymus-Dependent Antigen Sheep Red Blood Cells. J. Immunol. *199*, 2998–3003.

Menard, D., and Dondorp, A. (2017). Antimalarial Drug Resistance: A Threat to Malaria Elimination. Cold Spring Harb. Perspect. Med. *7*, a025619.

Nguyen, Q.N., Himes, J.E., Martinez, D.R., and Permar, S.R. (2016). The Impact of the Gut Microbiota on Humoral Immunity to Pathogens and Vaccination in Early Infancy. PLOS Pathog. *12*, e1005997.

Nogaro, S.I., Hafalla, J.C., Walther, B., Remarque, E.J., Tetteh, K.K., Conway, D.J., Riley, E.M., and Walther, M. (2011). The breadth, but not the magnitude, of circulating memory B cell responses to P. falciparum increases with age/exposure in an area of low transmission. PLOS ONE *6*, e25582.

Nojima, T., Haniuda, K., Moutai, T., Matsudaira, M., Mizokawa, S., Shiratori, I., Azuma, T., and Kitamura, D. (2011). In-vitro derived germinal centre B cells differentially generate memory B or plasma cells in vivo. Nat. Commun. *2*, 465.

Nonvignon, J., Aryeetey, G.C., Malm, K.L., Agyemang, S.A., Aubyn, V.N., Peprah, N.Y., Bart-Plange, C.N., and Aikins, M. (2016). Economic burden of malaria on businesses in Ghana: a case for private sector investment in malaria control. Malar. J. *15*, 454.

Obeng-Adjei, N., Portugal, S., Tran, T.M., Yazew, T.B., Skinner, J., Li, S., Jain, A., Felgner, P.L., Doumbo, O.K., Kayentao, K., et al. (2015). Circulating Th1-Cell-type Tfh Cells that Exhibit Impaired B Cell Help Are Preferentially Activated during Acute Malaria in Children. Cell Rep. *13*, 425–439.

Oh, J.Z., Ravindran, R., Chassaing, B., Carvalho, F.A., Maddur, M.S., Bower, M., Hakimpour, P., Gill, K.P., Nakaya, H.I., Yarovinsky, F., et al. (2014). TLR5-mediated sensing of gut microbiota is necessary for antibody responses to seasonal influenza vaccination. Immunity *41*, 478–492.

Osier, F.H., Fegan, G., Polley, S.D., Murungi, L., Verra, F., Tetteh, K.K., Lowe, B., Mwangi, T., Bull, P.C., Thomas, A.W., et al. (2008). Breadth and magnitude of antibody responses to multiple Plasmodium falciparum merozoite antigens are associated with protection from clinical malaria. Infect. Immun. *76*, 2240–2248.

Pérez-Mazliah, D., Ng, D.H., Freitas do Rosário, A.P., McLaughlin, S., Mastelic-Gavillet, B., Sodenkamp, J., Kushinga, G., and Langhorne, J. (2015). Disruption of IL-21 signaling affects T cell-B cell interactions and abrogates protective humoral immunity to malaria. PLOS Pathog. *11*, e1004715.

Pérez-Mazliah, D., Nguyen, M.P., Hosking, C., McLaughlin, S., Lewis, M.D., Tumwine, I., Levy, P., and Langhorne, J. (2017). Follicular Helper T Cells are Essential for the Elimination of Plasmodium Infection. EBioMedicine *24*, 216–230.

Phyo, A.P., Ashley, E.A., Anderson, T.J.C., Bozdech, Z., Carrara, V.I., Sriprawat, K., Nair, S., White, M.M., Dziekan, J., Ling, C., et al. (2016). Declining Efficacy of Artemisinin Combination Therapy Against P. Falciparum Malaria on the Thai-Myanmar Border (2003-2013): The Role of Parasite Genetic Factors. Clin. Infect. Dis. *63*, 784–791.

Rosser, E.C., Oleinika, K., Tonon, S., Doyle, R., Bosma, A., Carter, N.A., Harris, K.A., Jones, S.A., Klein, N., and Mauri, C. (2014). Regulatory B cells are induced by gut microbiota-driven interleukin-1β and interleukin-6 production. Nat. Med. *20*, 1334–1339.

Ryg-Cornejo, V., Ioannidis, L.J., Ly, A., Chiu, C.Y., Tellier, J., Hill, D.L., Preston, S.P., Pellegrini, M., Yu, D., Nutt, S.L., et al. (2016). Severe Malaria Infections Impair Germinal Center Responses by Inhibiting T Follicular Helper Cell Differentiation. Cell Rep. *14*, 68–81.

Shreiner, A.B., Kao, J.Y., and Young, V.B. (2015). The gut microbiome in health and in disease. Curr. Opin. Gastroenterol. *31*, 69–75.

Sprouse, M.L., Bates, N.A., Felix, K.M., and Wu, H.J. (2019). Impact of gut microbiota on gut-distal autoimmunity: a focus on T cells. Immunology *156*, 305–318.

Stern, J.N., Yaari, G., Vander Heiden, J.A., Church, G., Donahue, W.F., Hintzen, R.Q., Huttner, A.J., Laman, J.D., Nagra, R.M., Nylander, A., et al. (2014). B cells populating the multiple sclerosis brain mature in the draining cervical lymph nodes. Sci. Transl. Med. *6*, 248ra107.

Taylor, J.J., Pape, K.A., and Jenkins, M.K. (2012). A germinal center-independent pathway generates unswitched memory B cells early in the primary response. J. Exp. Med. *209*, 597–606.

Teng, F., Klinger, C.N., Felix, K.M., Bradley, C.P., Wu, E., Tran, N.L., Umesaki, Y., and Wu, H.J. (2016). Gut Microbiota Drive Autoimmune Arthritis by Promoting Differentiation and Migration of Peyer's Patch T Follicular Helper Cells. Immunity *44*, 875–888.

Tomayko, M.M., Steinel, N.C., Anderson, S.M., and Shlomchik, M.J. (2010). Cutting edge: hierarchy of maturity of murine memory B cell subsets. J. Immunol. *185*, 7146–7150.

van der Pluijm, R.W., Imwong, M., Chau, N.H., Hoa, N.T., Thuy-Nhien, N.T., Thanh, N.V., Jittamala, P., Hanboonkunupakarn, B., Chutasmit, K., Saelow, C., et al. (2019). Determinants of dihydroartemisinin-piperaquine treatment failure in Plasmodium falciparum malaria in Cambodia, Thailand, and Vietnam: a prospective clinical, pharmacological, and genetic study. Lancet Infect. Dis. *19*, 952–961.

Villarino, N.F., LeCleir, G.R., Denny, J.E., Dearth, S.P., Harding, C.L., Sloan, S.S., Gribble, J.L., Campagna, S.R., Wilhelm, S.W., and Schmidt, N.W. (2016). Composition of the gut microbiota modulates the severity of malaria. Proc. Natl. Acad. Sci. USA *113*, 2235–2240.

Weiss, G.E., Traore, B., Kayentao, K., Ongoiba, A., Doumbo, S., Doumtabe, D., Kone, Y., Dia, S., Guindo, A., Traore, A., et al. (2010). The Plasmodium falciparum-specific human memory B cell compartment expands gradually with repeated malaria infections. PLOS Pathog. *6*, e1000912.

World Health Organization (2018). World malaria report. https://apps.who.int/iris/bitstream/handle/10665/275867/9789241565653-eng.pdf.

World Health Organization (2019). World malaria report. https://www.who.int/publications/i/item/9789241565721.

Yaari, G., and Kleinstein, S.H. (2015). Practical guidelines for B-cell receptor repertoire sequencing analysis. Genome Med. *7*, 121.

Ye, J., Ma, N., Madden, T.L., and Ostell, J.M. (2013). IgBLAST: an immunoglobulin variable domain sequence analysis tool. Nucleic Acids Res. *41*, W34–W40.

Yilmaz, B., Portugal, S., Tran, T.M., Gozzelino, R., Ramos, S., Gomes, J., Regalado, A., Cowan, P.J., d'Apice, A.J., Chong, A.S., et al. (2014). Gut microbiota elicits a protective immune response against malaria transmission. Cell *159*, 1277–1289.

Yooseph, S., Kirkness, E.F., Tran, T.M., Harkins, D.M., Jones, M.B., Torralba, M.G., O'Connell, E., Nutman, T.B., Doumbo, S., Doumbo, O.K., et al. (2015). Stool microbiota composition is associated with the prospective risk of Plasmodium falciparum infection. BMC Genomics *16*, 631.

CellPress
OPEN ACCESS

STAR★METHODS

KEY RESOURCES TABLE

REAGENT or RESOURCE	SOURCE	IDENTIFIER
Antibodies		
CD45.2-APC, Clone 104	Biolegend	Cat# 109814; RRID:AB_389211
Ter119-APC/Cy7, Clone TER-119	Biolegend	Cat# 116223; RRID:AB_2137788
Dihydroethidium	Sigma Aldrich	Cat# 37291
Hoechst 33342	Sigma Aldrich	Cat# 875756-97-1; RRID:AB_10626776
FC block; anti-CD16/32, clone 2.4G2	BD Bioscience	Cat# 553142; RRID:AB_394657
CD3-FITC clone 145-2C11	Biolegend	Cat# 100305; RRID:AB_312670
CD4-PE-Cy7, clone RM4-5	Biolegend	Cat# 100527; RRID:AB_312728
CD4-PE, clone RM4-4	Biolegend	Cat# 116005; RRID:AB_313690
CD11a-FITC, clone M17/4	Biolegend	Cat# 101106; RRID:AB_312779
CD49d-PE, clone R1-2	Biolegend	Cat# 103607; RRID:AB_313038
CXCR5-biotin, clone L138D7	Biolegend	Cat# 145509; RRID:AB_2562125
Streptavidin-PE-Cy7	Biolegend	Cat# 405206; RRID:AB_10116480
CD44-FITC and APC-Cy7, clone IM7	Biolegend	Cat# 103021; RRID:AB_493684
PD-1 APC, clone 29F.1A12	Biolegend	Cat# 135209; RRID:AB_2251944
GL-7 Pac Blue, clone GL-7	Biolegend	Cat# 144613;RRID:AB_2563291
CD19 PerCP-Cy5.5, clone 6D5	Biolegend	Cat# 115534; RRID:AB_313654
CD138-BV421, Clone 281-2	Biolegend	Cat# 142507; RRID:AB_11204257
IgD APC-Cy7, clone 11-26c2a	Biolegend	Cat# 405716; RRID:AB_10662544
CD95-PE, clone SA367H8	Biolegend	Cat# 152608; RRID:AB_2632902
CD73-BV605, clone TY/11.8	Biolegend	Cat# 127215; RRID:AB_2561528
CD71-BV421, clone RI7217	Biolegend	Cat# 113813; RRID:AB_10899739
CD80-PE, clone 90	Biolegend	Cat# 104707; RRID:AB_313128
Bcl-6 PE-CF594, clone K112-91	BD Biosciences	Cat# 562401; RRID:AB_11152084
B220-AF488, clone RA3-6B2	ThermoFisher	Cat# 53-0452-82; RRID:AB_469907
CD3-AF594, clone 17A2	Biolegend	Cat# 100240; RRID:AB_2563427
GL7-AF647, clone GL7	BD Biosciences	Cat# 561529; RRID:AB_10716056
Peroxidase Affini Pure Goat Anti-Mouse IgM	Jackson ImmunoResearch	Cat# 115-035-020; RRID:AB_2338502
Peroxidase Affini Pure Goat Anti-Mouse IgG1	Jackson ImmunoResearch	Cat# 115-005-205; RRID:AB_2338461
Peroxidase Affini Pure Goat Anti-Mouse IgG2b	Jackson ImmunoResearch	Cat# 115-005-207; RRID:AB_2338463
Peroxidase Affini Pure Goat Anti-Mouse IgG2c	Jackson ImmunoResearch	Cat# 115-005-208; RRID:AB_2338464
Peroxidase Affini Pure Goat Anti-Mouse IgG3	Jackson ImmunoResearch	Cat# 115-005-209; RRID:AB_2338465
Goat anti-mouse HRP	Invitrogen	Cat# G21040; RRID:AB_2536527
Anti-CD40L mAB (MR1)	BioXcell	Cat# BE0017-1; RRID:AB_1107601
Armenian hamster IgG	BioXcell	Cat# BE0091; RRID:AB_1107773
Biological Samples		
Citrated Sheep Red Blood cells	HemoStat Laboratories; McAllister et al., 2017	N/A

(*Continued on next page*)

Continued

REAGENT or RESOURCE	SOURCE	IDENTIFIER
Chemicals, Peptides, and Recombinant Proteins		
Recombinant *Plasmodium yoelii* yP.y MSP1-10(XL)/VQ1, MRA-48 (MSP1$_{19}$)	BEI Resources Repository/MR4/ATC, NIAID, DC Kaslow	N/A
pHrodo Bioparticles	ThermoFisher	Cat# P35380
Critical Commercial Assays		
FoxP3 Transcription Staining Buffer Set	Thermofisher	Cat# 00-5523-00 \00-
RNeasy Mini Kit	QIAGEN	Cat# 74104
SMARTer Mouse BCR Profiling Kit	Takara Bio	Cat# 634422
Agilent 2100 Bioanalyzer High Sensitivity DNA Assay Kit	Agilent	Cat# 5067-4626
600-Cycle MiSe1 Reagent Kit v3	Illumina	Cat# MS-102-3003
Bradford Protein Assay	ThermoFisher	Cat# 23200
Microtainer Serum Separator Tubes Gold	Henry Schein Animal Health	Cat# 055955
Deposited Data		
Raw Sequence Reads	Generated for this paper	BioProject: PRJNA675100
Experimental Models: Cell Lines		
Plasmodium yoelii 17XNL	BEI Resources Repository/MR4/ATCC, NIAID, DC Kaslow	N/A
Plasmodium berghei ANKA	BEI Resources Repository/MR4/ATCC, NIAID, DC Kaslow	N/A
Experimental Models: Organisms/Strains		
C57BL/6N Conventional mice	Taconic Biosciences	Barrier room; IBU504
C57BL/6N Conventional mice	Charles River Laboratories	Barrier room; R01
C57BL/6N Germ Free mice	Taconic Biosciences	N/A
C57BL/6N Germ Free mice	Charles River Laboratories	N/A
Oligonucleotides		
Primer for IgM (First round PCR, 5′-CAGATCCCTGTGAGTCACAG TACAC-3, 12 μm; second round PCR, 5′-AATGATACGGCGACCA CCGAGATCTACACTATAGCCTA CACTCTTTCCCTACACGACGCT CTTCCGATCTNNNNNNNNGGG AAGACATTTGGGAAGGACTG AC-3′, 12.5 μM)	This paper	N/A
Software and Algorithms		
Immcantation Pipeline	Yaari and Kleinstein, 2015	https://immcantation.readthedocs.io/en/stable/
Presto software package	Stern et al., 2014	https://prestodb.io
IgBlast	Ye et al., 2013	https://www.ncbi.nlm.nih.gov/igblast/
ImMunoGeneTics Information System database	Giudicelli et al., 2006	http://www.imgt.org
Change-O	Gupta et al., 2015	https://changeo.readthedocs.io/en/stable/
Alakazam package	Stern et al., 2014	https://cran.r-project.org/web/packages/alakazam/index.html
Graphpad Prism 7	GraphPad	https://www.graphpad.com/scientific-software/prism/
Other		
NIH-31 Modified Open Formula Mouse/Rat Irradiated Diet	Envigo	Cat# 7913

CellPress
OPEN ACCESS

RESOURCE AVAILABILITY

Lead Contact

Further information and requests for resources and reagents should be directed to and will be fulfilled by the Lead Contact, Nathan W. Schmidt (nwschmid@iu.edu).

Materials Availability

This study did not generate new unique reagents.

Data and Code Availability

The expressed antibody gene sequencing dataset generated from mice in this study is publicly available and deposited in the NCBI Sequence Read Archive Original data have been deposited to BioProject: PRJNA675100.

EXPERIMENTAL MODEL AND SUBJECT DETAILS

Mice

Conventional female C57BL/6N mice (6 to 8 weeks old) were purchased from Charles River Laboratories (Wilmington, MD) and Taconic Biosciences (Hudson, NY). Mice were allowed to acclimate a minimum of 5 days prior to start of experiments. Germ-free female C57BL/6N mice (6-8 weeks old) were purchased from Taconic Biosciences. Immediately upon arrival to the University of Louisville animal facility, GF mice were gavaged with 200uL cecal contents from a single donor diluted in saline for 1 to 3 consecutive days and stored in SPF conventional housing. Mice were allowed to acclimate 1 week prior to the start of experiments. All mice were fed NIH-31 Modified Open Formula Mouse/Rat Irradiated Diet and non-acidified water. All animal experiments were carried out at the University of Louisville and University of Indiana adhering to the local and national regulation of laboratory animal welfare, and all procedures were reviewed and approved by the university's Institutional Animal Care and Use Committees.

Plasmodium Infections

Mice were infected with *P. yoelii* 17XNL or *P. berghei* ANKA by intravenous injection of $1x10^5$ parasitized red blood cells (unless otherwise noted) prepared from either frozen/thawed stabilite or fresh donor blood. In secondary infection experiments, mice were infected with *P. yoelii* and parasitemia was monitored 4 days post infection until clearance (~15-20 days post infection). 60 days post *P. yoelii* infection, mice were infected with *P. berghei* and parasitemia was evaluated starting at day 4 post *P. berghei* infection (day 64 post initial infection) through parasite clearance or until endpoint was reached.

METHOD DETAILS

Evaluation of parasitemia

Blood samples were taken through tail snips at regular intervals ranging from days 4-30 post infection. Percent parasitemia evaluated as percent infected red blood cells per total red blood cells was assessed through blood smear or flow cytometry. For blood smear evaluation, fixed and giemsa stained thin blood smears, parasitized versus total red blood cells were counted at 1000x magnification. For flow cytometry evaluation, a blood droplet was added to PBS, fixed with 0.00625% gluteraldehyde, and stained with CD45.2-APC (clone 104; Biolegend, San Diego, CA), Ter119-APC/Cy7 (clone TER-119; Biolegend, Sand Diego, CA), dihydroethidium (Sigma Aldrich, St. Louis, MO), and Hoechst 33342 (Sigma Aldrich; St. Louis, MO). Parasitized red blood cells were defined as $CD45.2^-$ $Terr119^+$ $Dihydroethidium^+$ $Hoechst^+$ cells.

Cellular immune response

Spleens were harvested from mice at the indicated day post infection and smashed through screens in RP-10 media to generate a single cell suspension. RP-10 was generated by supplementing Hyclone RPMI Medium 1640 (GIBCO, Thermo Fisher Scientific, Waltham, MA) with 10% fetal bovine serum (FBS) (Atlanta Biologicals, Inc., Lawrenceville, GA), 1.19 mg/ml HEPES (Thermo Fisher Scientific, Waltham, MA), 0.2 mg/ml L-glutamine (Research Products International Corp., Mt. Prospect, IL), (0.05 units/ml & 0.05 mg/ml) penicillin/streptomycin (Invitrogen, Grand Island, NY), 0.05 mg/ml gentamicin sulfate (Invitrogen, Grand Island, NY), and 0.05 μM 2-Mercaptoethanol (Thermo Fisher Scientific Inc., Waltham, MA). Single cell suspensions were treated with ammonium chloride potassium to lyse red blood cells. Cells were extracellularly stained for 30 minutes at 4°C with antibodies resuspended in FACS buffer (1x PBS, 0.02% sodium azide, and 1% FCS) with FC block (anti-CD16/32; clone 2.4G2), followed by fixation with fixation buffer (Biolegend, San Diego, CA). For biotinylated-CXCR5 staining cells were stained for 30 minutes at room temperature prior to staining with fluorescence-conjugated streptavidin.

For intracellular staining, staining was carried out using the eBioscience FoxP3/Transcription Factor Staining Buffer Set (ThermoFisher, Waltham, MA) according to manufacturer's instructions. All samples were collected using a BD LSRFortessa (BD Biosciences, San Jose, CA)) and analyzed by FlowJo software (Tree Star, Ashland, OR).

Antibodies

CD3-FITC clone 145-2C11, CD4-PE-Cy7 clone RM4-5, CD4-PE clone RM4-4, CD11a-FITC clone M17/4, CD49d-PE clone R1-2, CXCR5-biotin clone L138D7, PE-Cy7 streptavidin, CD44-FITC and CD44-APC-Cy7 clone IM7, PD-1 APC clone 29F.1A12, GL7-PacBlue and GL7-PE clone GL7, CD19-PerCP-Cy5.5 clone 6B2, CD138-BV421 clone 281-2, IgD-APC-Cy7 clone 11-26c2a, CD95-PE clone SA367H8, CD73-BV605 clone TY/11.8, CD71-BV421 Clone RI7217, and CD80-PE clone 90,were purchased from Biolegend (San Diego, CA). BcL6-PE-CF594 clone K112-91 was purchased from BD Biosciences, San Jose, CA)

Confocal Microscopy

Spleens were collected from mice at days 0, 7, 9, and 11, placed sequentially in a 10, 20, and 30% sucrose gradient overnight, and flash frozen in OCT. Spleens were sectioned in a cryostat and mounted on slides at ~8 um and stored at −80°C. Slides were blocked in 1% BSA diluted in PBS and stained overnight at 4° with B220-AF488 clone RA3-6B2 (ThermoFisher Scientific, Waltham, MA), CD3-AF594 clone 17A2 (Biolegend, San Diego, CA), and GL7-AF647 clone GL7 (BD Biosciences, San Jose, CA). Tissue was covered with ProLong Gold antifade reagent (Thermo Fisher Scientific, Waltham, MA) and imaged using a Leica TCS SP8 confocal microscope. Images were analyzed using Fiji ImageJ software for MAC.

ELISA

For detection of *P. yoelii* $MSP1_{19}$ specific antibodies, blood was collected through retro-orbital bleed on the indicated days post infection, allowed to clot for at least 30 minutes, and centrifuged at 4° for 10 minutes at 10,000 rpm to collect serum. Serum was stored at −80°C. MaxiSorp Immuno plates (Thermo Fisher Scientific, Waltham, MA) were coated with 1 ug/ml recombinant $MSP1_{19}$ (obtained through the MR4 as part of the BEI Resources Repository, NIAID, NIH: *Plasmodium yoelii* yP.y MSP1-10(XL)/VQ1, MRA-48, deposited by DC Kaslow). Dilutions of serum were added to wells and total $MSP1_{19}$-specific IgM, IgG1, IgG2b, IgG2c, and IgG3 antibodies were detected with horseradish peroxidase-conjugated goat anti-mouse IgM, IgG1, IgG2b, IgG2c, and IgG3 (Jackson ImmunoResearch, West Grove, PA) followed by 3,3′,5,5′-tetramethylbenzidine substrate (Arcos Organics). 2M H_2SO_4 was used to halt the reaction and plates were read using a microplate reader with the absorbance at 450 nm.

To evaluate bulk whole parasite lysate specific IgG, *Plasmodium* whole parasites were isolated from whole blood of *Plasmodium yoelii* infected mice using a 35%/65% percoll gradient. Infected red blood cells were than lysed and protein concentration was calculated using a Bradford Protein Assay (ThermoFisher Scientific, Waltham, MA). MaxiSorp Immuno plates (Thermo Fisher Scientific, Waltham, MA) were coated with 10 μg/ml whole parasite lysate, a 1:200 dilution of serum was added to each well, and total parasite specific IgG was detected using horseradish peroxidase-conjugated goat anti-mouse IgG antibody (Jackson ImmunoResearch, West Grove, PA)

Expressed Antibody Repertoire Sequencing and Analysis

Spleens were removed from *P. yoelii* infected mice at day 10 post infection, a single cell suspension was generated, cells were extracellularly stained for 30 minutes at 4°, and then cells were resuspended in FACS buffer. Naive B cells and GCB cells were sorted using a BD FACSAria III (BD Biosciences, San Jose, CA). 100,000 B cells were sorted per cell type, per mouse. RNA was extracted from the cells using the RNeasy Mini Kit (QIAGEN, Hilden, Germany) according to manufacturer's instruction. Naive B cell IgM and GCB IgG/IgM 5′RACE AIRR-seq libraries were generated using the SMARTer Mouse BCR Profiling Kit (Takara Bio, Cat. No. 634422; Mountain View, CA, USA), following the manufacturer's instructions. Because only IgG oligos for generating nested PCR amplicons were available in the kit, additional custom IgM primers positioned in the CH3 and CH1 regions of IGHM were developed using available genomic sequence (first round PCR, 5′-CAGATCCCTGTGAGTCACAGTACAC-3′, 12 μm; second round PCR, (5′-AATGATACGGCG ACCACCGAGATCTACACTATAGCCTACACTCTTTCCCTACACGACGCTCTTCCGATCTNNNNNNNNGGGAAGACATTTGGGAAGG ACTGAC-3′, 12.5 μM). The quality of individual barcoded IgM and IgG libraries were assessed using the Agilent 2100 Bioanalyzer High Sensitivity DNA Assay Kit (Agilent, Cat. No. 5067-4626). Libraries were then pooled to 10 nM and sequenced on the Illumina MiSeq platform using the 600-cycle MiSeq Reagent Kit v3 (2x300 bp, paired-end; Illumina, Cat. No. MS-102-3003).

BCR Rep Sequencing Processing and Analysis

(PrestoR (upfront QC metrics and trimming plots), Presto, Change-O, R-Analysis Pipeline)

Following library demultiplexing, repertoire sequencing data were processed end-to-end using tools/software within the Immcantation pipeline (Yaari and Kleinstein, 2015). Library metrics for each sample at each processing step are provided in Table S1. Using the Presto software package (Stern et al., 2014), all reads in each library were trimmed to Q = 20 using the Filterseq trimqual sub command in order to avoid erroneous merging of paired-end reads. IgM/IgG primer sequences were identified among the reads using maskPrimers align and removed, and those reads lacking IgM/IgG primer sequences were excluded from further analysis. Paired-end reads were assembled using pairseq allowing for a maximum overlap error rate of 0.1. For each read pair, *de novo* assembly was attempted first using assemblePairs sequential, and in cases for which bp overlaps were insufficient, blastn guided assembly was utilized. Resultant assembled sequences < 400 bp were excluded with the filterSeq length sub command. Duplicate sequences within each library were then collapsed using collapseSeq and the "dupcount" was recorded; sequences with 'dupcount' < 2 were excluded using splitSeq. Assignments of processed sequences to germline IGHV, IGHD, and IGHJ genes/alleles were determined utilizing IgBlast (Ye et al., 2013)and C57BL/6 germline sequences available in the ImMunoGeneTics Information System

(IMGT) database (Downloaded June 2019)(Giudicelli et al., 2006); sequence for the gene IGHV1-2 was also manually added as it was not present in the downloaded version of the IMGT database. Using Change-O (Gupta et al., 2015), sequences within each animal, across isotype and cell subset libraries were assigned to clones using the DefineClones function, based on IGHV/J gene assignments and junction sequence similarity (Hamming distance = 0.15). The relative abundance of clones was calculated and compared at the level of sample and vendor using the estimateAbundance function in the Alakazam package (Stern et al., 2014); these are based on abundance distribution methods as described in Chao et al. (2014, 2015). From the same package, the repertoire diversity (Hill, 1973) as calculated and compared at the sample level and at the vendor level using the alphaDiversity function. To account for variation in sampling read depth, both of these functions were implemented using bootstrapping with 200 resamplings per repertoire. Using the countGenes function within in the Alakazam package (Stern et al., 2014), gene usage (IGHV and IGHJ) was estimated based on gene segment representation among unique sequences specific to each isotype/subset within a sample. The RDI (repertoire dissimilarity index) (Bolen et al., 2017), which quantifies pairwise differences in gene segment usage, was calculated at the vendor level for each cell subset and isotype using the calcRDI function. To complement RDI analysis, principal component analysis was also employed using clone-based gene usage frequency estimates within each cell subset and isotype. Finally, the Kruskal-Wallace test was used to test for group differences (Tac versus CR) in IGHV and IGHJ gene usage within each cell subset and isotype. A multiple testing correction was implemented (false-discovery rate, FDR) using the Benjamini-Hochberg method (Benjamini and Hochberg, 1995). The Kruskal-Wallace test was also used to assess between group differences in the diversity metric at $q = 2$ (the Simpson's index) and RDI ($p < 0.05$).

Western Blot

Plasmodium whole parasites were isolated from whole blood of *Plasmodium yoelii* infected mice using a 35%/65% percoll gradient. Infected red blood cells were than lysed and protein concentration was calculated using a Bradford Protein Assay (ThermoFisher, Waltham, MA 23200). 20 μg of *Py* parasite lysate were separated on 4%–12% bis-tris SDS-PAGE mini gels (Bolt^{TM}, ThermoFisher Scientific, Waltham, MA), transferred to PVDF membranes using the iBlot2^{TM} Dry Blotting System (ThermoFisher Scientific, Waltham, MA), stained with Ponceau S (0.1% w/v of 5% glacial acetic acid), cut, rinsed with 0.1M NaOH to remove the Ponceau and blocked overnight in 5% BSA-TBS-T 1x. The membranes were then probed against individual mouse serum (1:5000, overnight) and goat anti-mouse HRP (1:10000, H^{+}L, Invitrogen G21040, 45 minutes). Development was performed using Clarity ECL (Bio-Rad) and acquisition using ChemiDoc^{TM} MP (Bio-Rad).

Antibody treatments

Mice were treated intravenously with 0.43 mg anti-CD40L mAb (MR1) (BioXcell, West Lebanon, NH) or 0.42 mg Armenian hamster IgG (BioXcell, West Lebanon, NH) at days 3, 5, 7, 9, 11, and 13.

Evaluation of Iron Levels

Blood was collected from resistant and susceptible mice via cardiac puncture from CO_2 asphyxiated mice and placed it in Microtainer Serum Separator Tubes (SST) Gold (Henry Schein Animal Health; #055955). Blood was allowed to clot for 30 minutes and spun at 4°C for 10 minutes at 10,000 rpm. Serum was pooled from groups of two mice and shipped on dry ice to Charles River for analysis.

Phagocytosis Assay

Spleens were removed from *P. yoelii* infected mice at days 7 and 14 p.i. and incubated with pHrodo Bioparticles (ThermoFisher Scientific, Waltham, MA). Cells were incubated for 1-2 hours at 37°C and flow cytometry was performed to evaluated pHrodo^{+} monocytes.

Sheep Red Blood Cell Injections

Citrated sheep red blood cells (HemoStat Laboratories, Dixon, CA) were prepared following the protocol described by McAllister et al. (2017). Mice were injected intraperitoneally with 150 μL of 1:10 dilution of SRBCs. Spleens were removed prior to immunization and at days 5, 7, 9, and 14 post immunization.

QUANTIFICATION AND STATISTICAL ANALYSIS

Statistical analyses were performed using Graphpad Prism 7 software (GraphPad Software, La Jolla, CA). Specific analyses and statistical significance are described in individual figure legends.

Cell Reports, Volume *33*

Supplemental Information

Gut Microbiota Composition Modulates the Magnitude and Quality of Germinal Centers during *Plasmodium* Infections

Morgan L. Waide, Rafael Polidoro, Whitney L. Powell, Joshua E. Denny, Justin Kos, David A. Tieri, Corey T. Watson, and Nathan W. Schmidt

Supplemental Figure 1

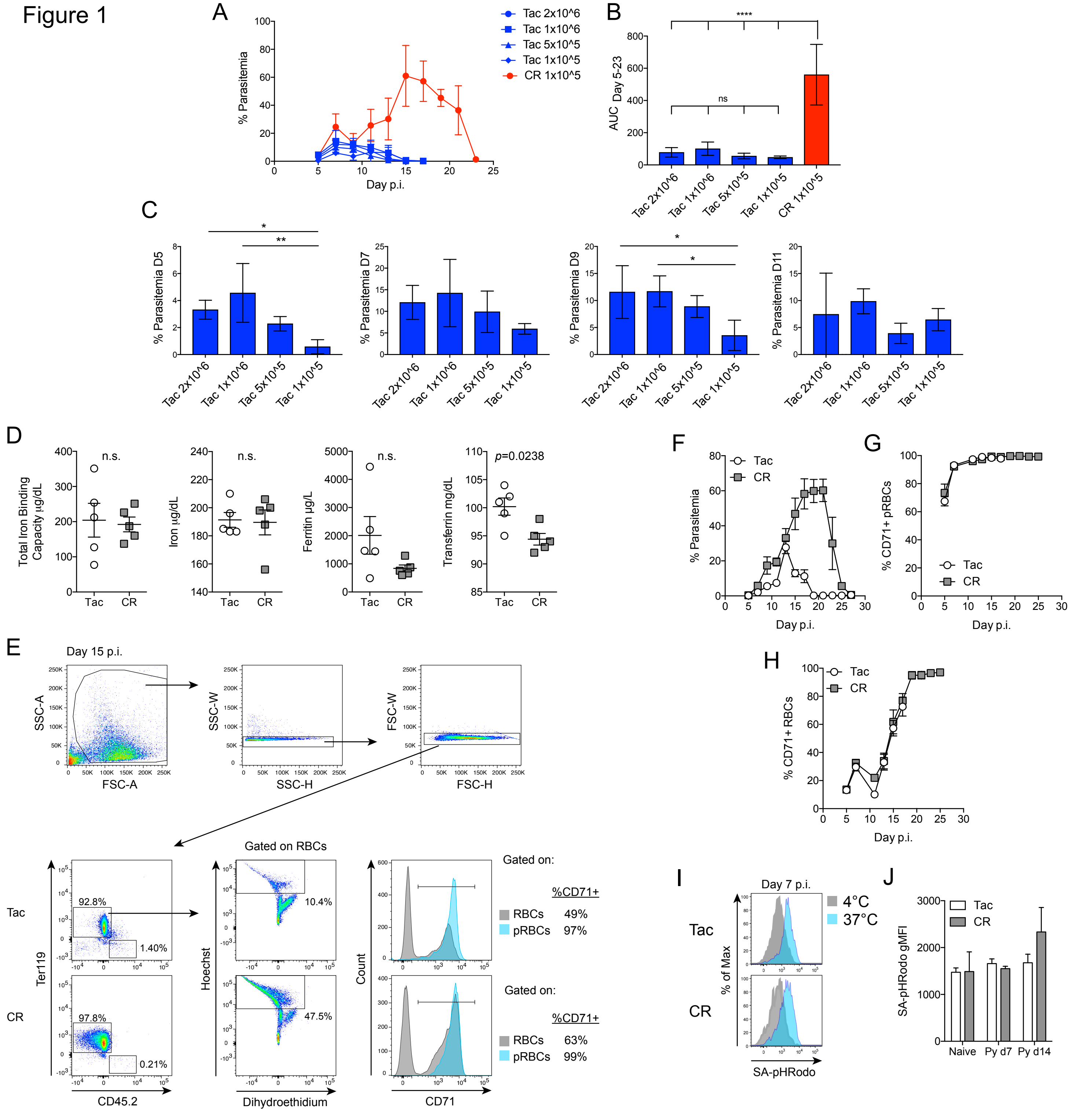

Supplemental Figure 1. Differences in parasite burden not influenced by parasite dose, differential iron levels, erythropoiesis, or macrophage phagocytosis. Related to Figure 1. Resistant Tac mice were infected with increasing doses of *P. yoelii* infected red blood cells. (A) Parasitemia of Tac mice compared to CR mice (n=4). (B) Area under the curve analysis of parasitemia curves. (C) Bar graphs showing parasitemia in Tac mice individually at days 5-11. Data (mean±S.D) were analyzed by One-way ANOVA with multiple comparisons. (D) Results from serum iron panel analysis. Data (mean±S.E.) are from 5 samples (each sample is pooled sera from 2 mice) and were analyzed by Mann-Whitney test. n.s. = not significant. (E-H) Mice from Tac and CR were infected with pRBCs. (E) Representative FACS plots displaying gating strategy for RBCs (Ter-119+, CD45.2-) and pRBCs (Ter-119+, CD45.2-, Hoechst 33342+, dihydroethidium+) that are CD71+. (F) Percent parasitemia. (G) Percent of pRBCs that are CD71+. (H) Percent of total RBCs that are CD71+. Data (mean±S.E.) from 3 mice per group are representative of 3 experiments. (I-J) Phagocytic activity of CD3-CD11bhiCD11c- splenocytes from uninfected or infected mice. (I) Representative histogram of pHrodo® Red expression in CD3-CD11bhiCD11c- splenocytes harvested from day 7 P. yoelii-infected mice following a 1- to 2-hour incubation at the indicated temperature. (J) pHrodo® Red geometric fluorescence intensity (gMFI) in CD3-CD11bhiCD11c- splenocytes after a 1- to 2-hour incubation at 37°C. Data (mean+S.E.) are from 3 mice per group and representative of 2 experiments.

Supplemental Figure 2

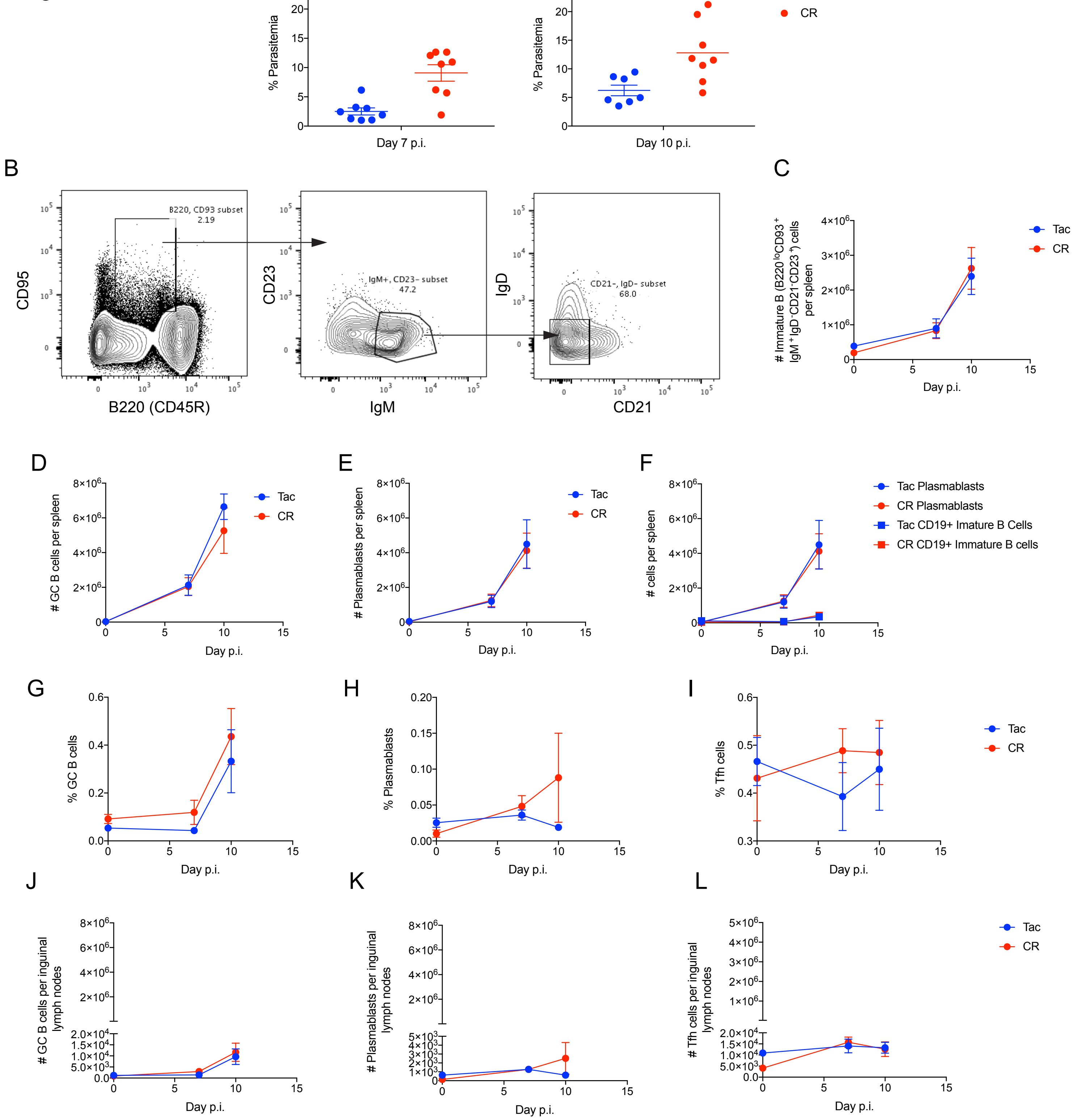

Supplemental Figure 2. Parasite burden in Tac and CR mice is not associated with differential numbers of splenic immature B cells or lymph node Tfh cells, plasmablasts, or GC B cells. Related to Figure 1. Tac and CR mice were infected with *P. yoelii* and spleens and inguinal lymph nodes were removed at days 0, 7, and 10 p.i. (A) Percent parasitemia of mice at the indicated time points. (B) Gating strategy for immature B cells (Ueda, Liao et al. 2007). (C) Numbers of immature B cells (B220loCD93+IgM+IgD-CD21-CD23-), (D) GC B cells (CD19+CD95+GL7+), and (E) plasmablasts (CD19+B220+IgD-CD138+) per spleen. (F) To show that very few immature B cells are incorporated into the plasmablasts staining panel, immature B cells were gated on CD19 and the total number of CD19+ immature B cells and Plasmablasts per spleen were shown on the same graph. (D-I) The frequencies and total cell numbers of Tfh cells (G, J), Plasmablasts (H,K), and GC B cells (I, L) in the inguinal lymph nodes are shown. Data (mean±S.E.) are from two independent experiments with n=8 mice per timepoint.

Supplemental Figure 3

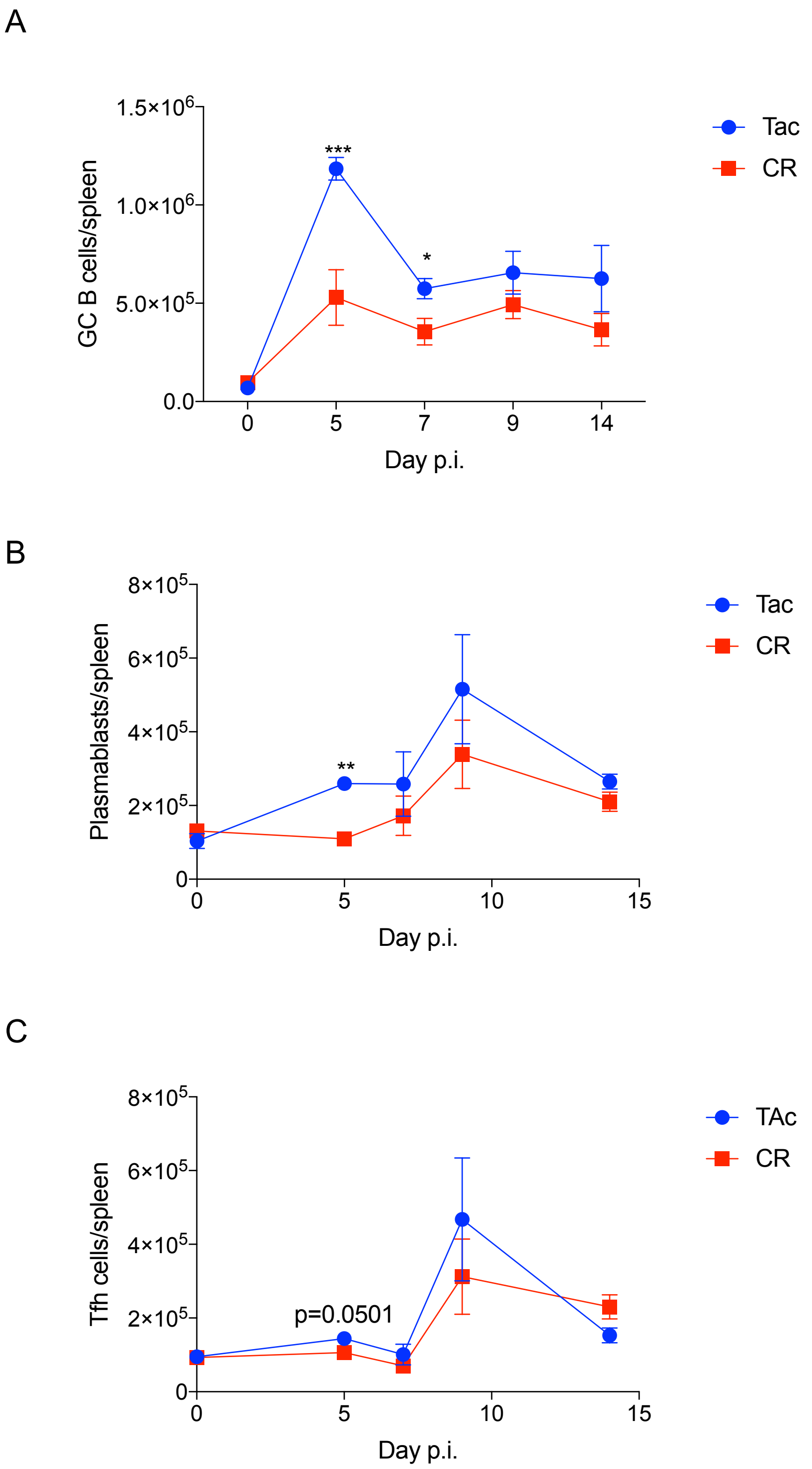

Supplemental Figure 3. Tac mice immunized with sheep red blood cells exhibit increased GC B cell response compared to immunized CR mice. Related to Figure 1. Tac and CR mice received a single I.P. injection of 150 uL citrated sheep red blood cells. Spleens were removed at days 0, 5, 7, 9, and 14. GC B cell (A), plasmablast (B), and Tfh cell (C) numbers per spleen were analyzed. Data (mean±S.E.) are from two independent experiments (n=3-7 mice per timepoint) and were analyzed by unpaired T test * $p < 0.05$, ** $p < 0.01$, *** $p < 0.001$.

Supplemental Figure 4

A

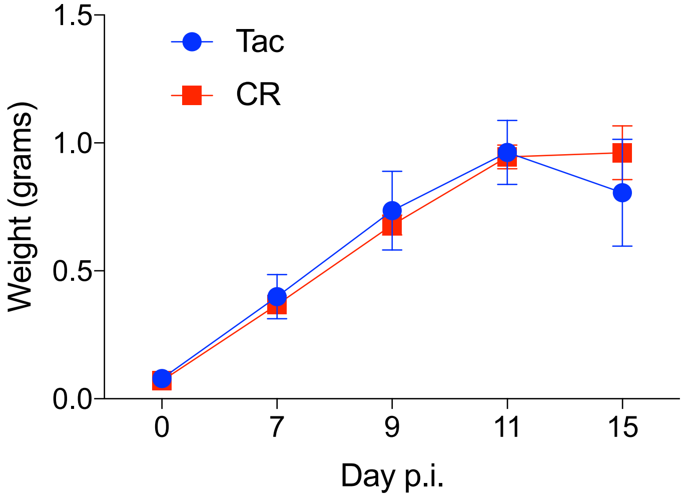

B

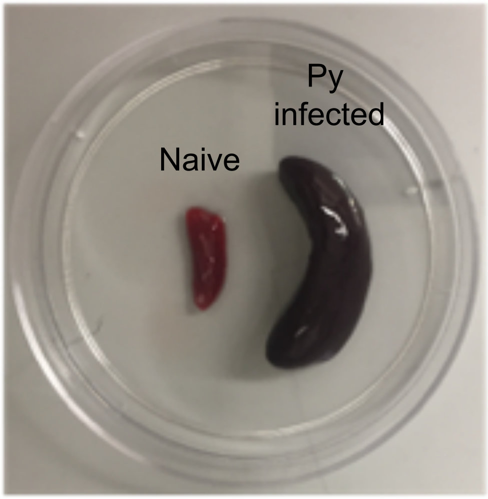

C

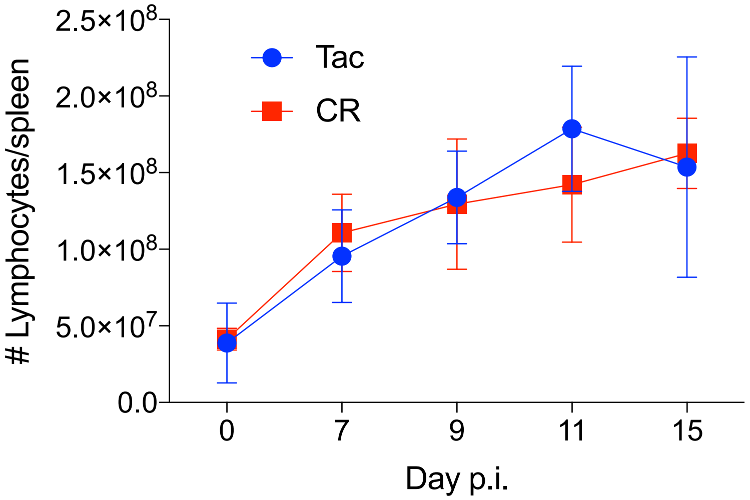

Supplemental Figure 4. Splenic expansion occurs equally in both resistant and susceptible mice. Related to Figure 3. Spleens were removed from both Tac and CR mice between days 0 and 15 post *P. yoelii* infection. (A) Spleen weights were recorded at each timepoint. (B) Comparison of naïve spleen (left) compared to infected spleen (right) to illustrate change in size and color post *Plasmodium* infection. (C). Number of lymphocytes per spleen were calculated using a hemocytometer at each timepoint. Data (mean+S.E.) are from 3 separate experiments.

Supplemental Figure 5

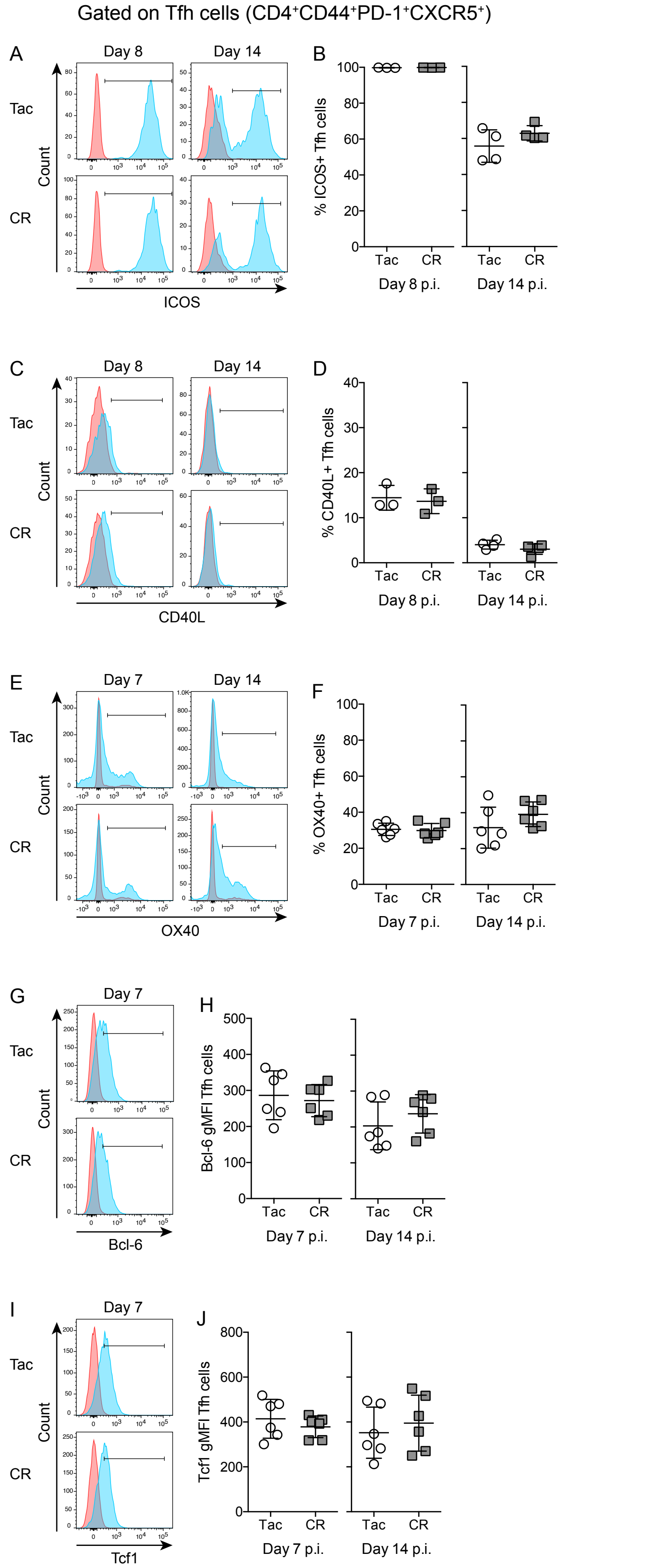

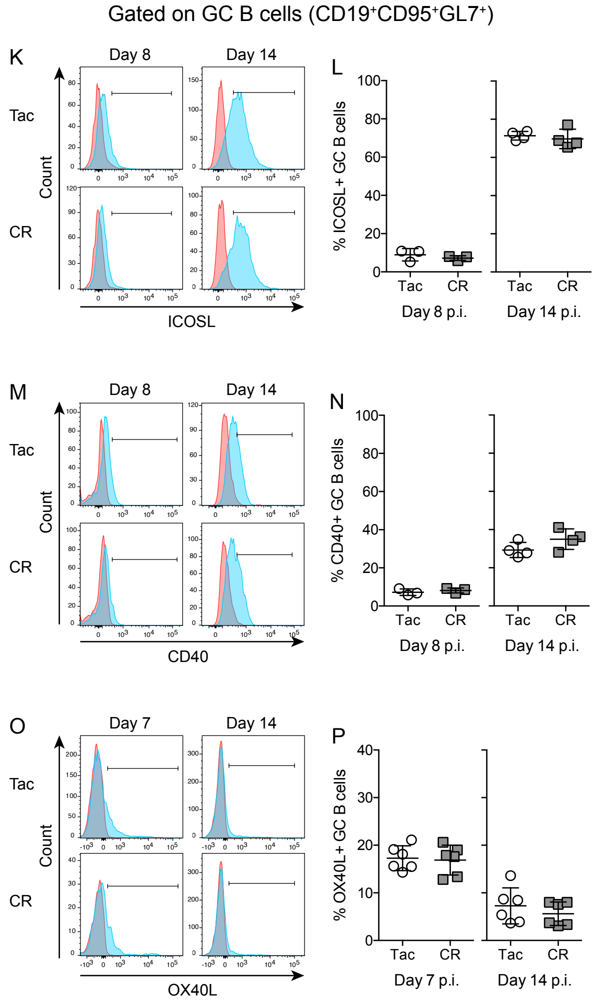

Supplemental Figure 5. Phenotypic analysis of co-stimulatory receptors and transcription factors on Tfh cells and GC B cells. Related to Figure 4. Tac and CR mice were infected with *P. yoelii* and spleens were removed on the indicated day post infection. Flow cytometry analysis on Tfh cells (CD4+CD44+PD-1+CXCR5+) for ICOS (A-B), CD40L (C-D), OX40 (E-F), Bcl-6 (G-H), or TCf1 (I-J). Flow cytometry analysis on GC B cells (CD19+CD95+GL7+) for ICOSL (K-L), CD40 (M-N), or OX40L (O-P). Data (mean+S.E.) are from 1 to 2 experiments.

Supplemental Figure 6

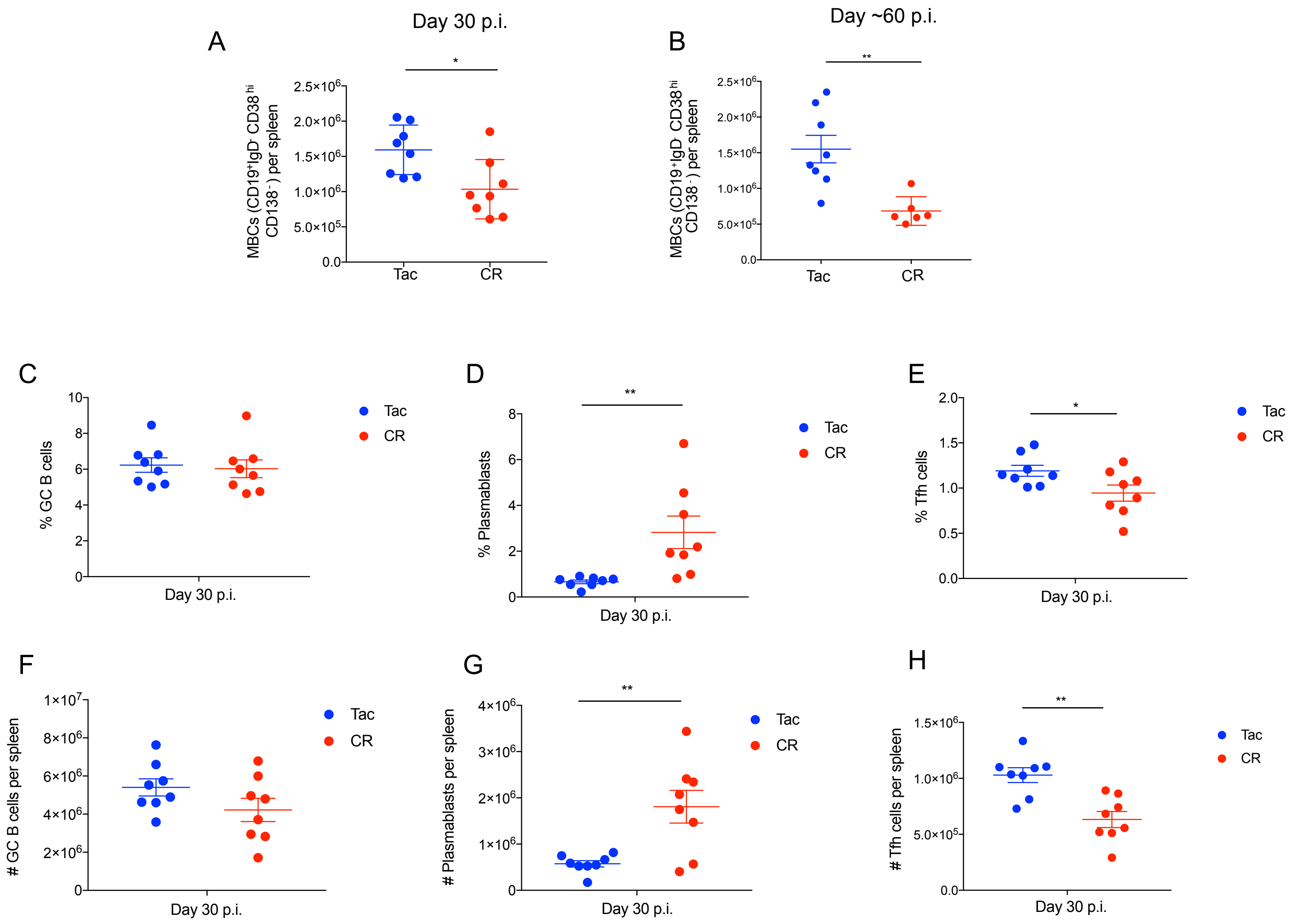

Supplemental Figure 6. Cellular analysis in the spleens of mice post *Plasmodium* clearance. Related to Figure 4. Tac and CR mice were infected with *P. yoelii*. Spleens were removed and cellular analysis done following clearance. The number of memory B cells (CD19+IgD-CD38hiCD138-) cells per spleen were determined at (A) day 30 and (B) day 60-62 p.i. The frequency and number of (C, F) GC B cells, (D, G) plasmablasts, and (E, H) Tfh cells were determined at day 30 post infection. Data (mean+S.E.) are from two separate experiments with n=6-8 mice per timepoint and were analyzed by unpaired T test * $p < 0.05$, ** $p < 0.01$

Supplemental Figure 7

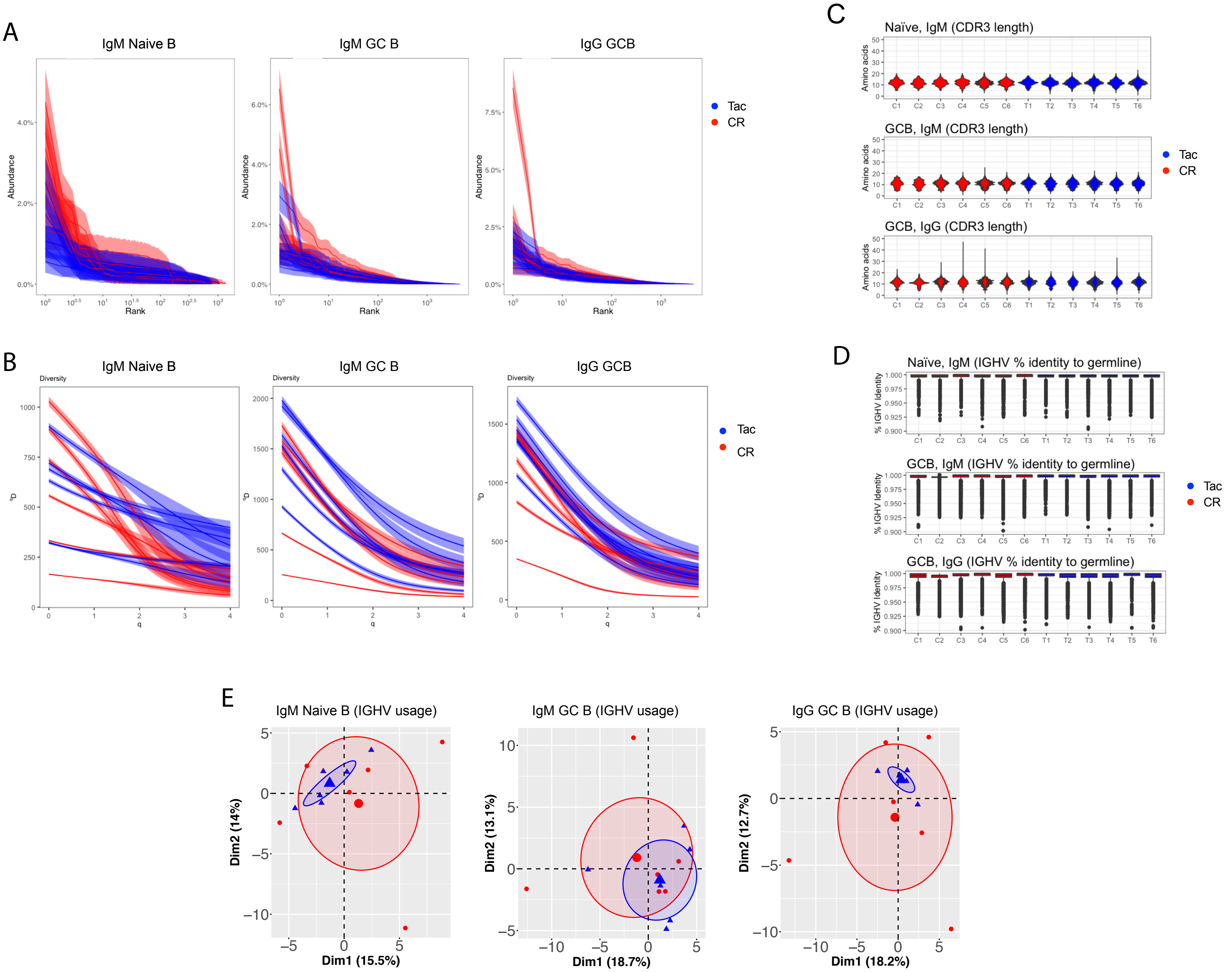

Supplemental Figure 7. Additional BCR analysis in Tac and CR naïve and GC B cells. Related to Figure 5. (A) Clonal abundance curves and (B) repertoire diversity curves at diversity orders, q=0-4, are shown for each animal for naïve cells, IgM+ GC B cells, and IgG+ GC B cells. Shading in plots represents the 95% confidence interval, as determined by 200 bootstrap resamplings of the data. (C) Variation in CDR3 amino acid length across all sequences in naïve cells, IgM+ GC B cells, and IgG+ GC B cells. (D) Variation in percent identity matches of each IGHV sequence to the closest germline IGHV gene. (E) Plots of the first two principle components associated with variation in IGHV gene usage in naïve cells, IgM+ GC B cells, and IgG+ GC B cells, each point representing an individual animal. Ellipses representing the 95% confidence interval around the centroid (large symbol) of each group are shown. Data for each animal are colored by group (red, CR, n=6; blue, Tac, n=6).

Supplemental Table 1. Repertoire sequencing library metrics, related to Figure 5. Repertoire sequencing library metrics for 6 Tac (T) mice and 6 CR (C) mice at the processing step of the analysis. Samples were processed end-to-end using tools/software within the Immcantation pipeline.

Sample	Subset	Isotype	Mask Primers	Filter Length	Collapse Seq
T1	GCB	IGM	287666	223545	87107
T1	GCB	IGG	566741	436494	150478
T1	N	IGM	98251	66908	20729
C1	GCB	IGM	181121	118500	40185
C1	GCB	IGG	235058	118377	41536
C1	N	IGM	135413	76497	24977
T2	GCB	IGM	224432	161007	54695
T2	GCB	IGG	328378	184456	63304
T2	N	IGM	258843	95458	28974
C2	GCB	IGM	234222	154403	41644
C2	GCB	IGG	224955	115217	33442
C2	N	IGM	143852	73206	20383
T3	GCB	IGM	644981	441600	151621
T3	GCB	IGG	480006	275387	100028
T3	N	IGM	217858	125158	41708
C3	GCB	IGM	871160	639836	223103
C3	GCB	IGG	708422	451758	157817
C3	N	IGM	131315	85139	31476
T4	GCB	IGM	1086665	702395	248925
T4	GCB	IGG	1015353	707352	247355
T4	N	IGM	706524	140944	46025
C4	GCB	IGM	528872	345868	124013
C4	GCB	IGG	183988	112431	45607
C4	N	IGM	323325	118636	38285
T5	GCB	IGM	278999	142960	55883
T5	GCB	IGG	141823	79810	32975
T5	N	IGM	968299	175663	56509
C5	GCB	IGM	1887600	1299232	439461
C5	GCB	IGG	1665908	964155	314222
C5	N	IGM	540189	266160	88439
T6	N	IGM	475287	228067	76141
C6	N	IGM	203445	83307	29945
T6	GCB	IGM	333549	211314	81241
T6	GCB	IGG	448507	255978	96530
C6	GCB	IGM	994200	639063	230509
C6	GCB	IGG	324214	176141	70687

Supplemental Table 2. (See data in excel document). Results of one-way ANOVA, related to Figure 5. Results of one-way ANOVA, assessing differential usage of IGHV and IGHJ gene between Tac and CR mice in naïve cells, IgM+ GC B cells, and IgG+ GC B cells. Both uncorrected P values and q values based on the false-discovery rate (Benjamini and Hochberg 1995) are provided.
